# Targeting the ARL4C/RAP1/PI3K-Akt-mTOR signaling loop promotes ARL4C ubiquitination and reverses oxaliplatin resistance in colorectal cancer

**DOI:** 10.7150/thno.117590

**Published:** 2026-01-01

**Authors:** Yang Wang, Zewen Chang, Ziquan Sun, Lu Wang, Jingtao Li, Guodong Li, Yuliuming Wang, Jinning Zhang, Lianjie Ai, Zhongxu Zhang, Jiaojiao Dong, Ming Liu

**Affiliations:** 1 Department of General Surgery, The Fourth Affiliated Hospital of Harbin Medical University, Harbin, China.; 2 Colorectal Oncology Surgery, The Second Affiliated Hospital of Harbin Medical University, Harbin, China.; 3 Heilongjiang Province Key Laboratory of Digestive Surgery and Nutrition & Metabolism, The Fourth Affiliated Hospital of Harbin Medical University, Harbin, Heilongjiang, China.

**Keywords:** ARL4C, oxaliplatin, β-Lapachone, colorectal cancer, ubiquitination

## Abstract

**Background:** Oxaliplatin resistance poses a significant therapeutic challenge in colorectal cancer (CRC), contributing to disease progression and poor clinical outcomes. There is an urgent need to identify novel molecular targets to overcome chemoresistance and inhibit metastatic dissemination.

**Methods:** We conducted integrative multi-omics analyses to identify genes associated with oxaliplatin resistance in CRC and detected ARL4C, a small GTPase, as a candidate driver. Functional experiments, including gene knockdown/overexpression, mutant construction, cell viability, apoptosis, migration, and invasion assays, as well as *in vivo* mouse models, were used to evaluate the role of ARL4C. Signaling pathways were examined using proteomics and molecular biology techniques. We employed network pharmacology and molecular docking to identify ARL4C-targeting compounds and selected β-Lapachone for further validation.

**Results:** ARL4C was significantly overexpressed in oxaliplatin-resistant CRC tissues and correlated with poor prognosis and increased metastatic potential. Mechanistic studies revealed that ARL4C activates RAP1/PI3K-Akt-mTOR and RAC1/Arp2/3 signaling axes, promoting cell survival, epithelial-mesenchymal transition, and invasion. ARL4C also inhibited its own ubiquitination by regulating USP38, forming a positive feedback loop that enhanced protein stability following chemotherapy. β-Lapachone was identified as a direct ARL4C inhibitor that binds competitively at the LYS128 residue, disrupting USP38 interactions and promoting ARL4C degradation. Combination therapy with β-Lapachone and oxaliplatin significantly suppressed tumor growth, reduced metastasis, reversed drug resistance, and mitigated oxaliplatin-induced renal toxicity in preclinical models.

**Conclusions:** Our study identifies ARL4C as a critical mediator of chemoresistance and metastasis in CRC. Targeting ARL4C with β-Lapachone restores oxaliplatin sensitivity and enhances therapeutic efficacy, offering a promising combinatorial strategy with strong potential for clinical translation in drug-resistant CRC.

## Introduction

Colorectal cancer (CRC) is among the most prevalent and lethal malignancies of the digestive system worldwide. According to GLOBOCAN 2022 data, CRC ranks third in global incidence and second in cancer-related mortality, with an alarming trend toward early onset 1, 2. It has become a significant public health concern threatening human health. While early-stage CRC patients can achieve favorable outcomes through surgical resection, the majority are diagnosed at advanced stages, requiring multimodal interventions including chemotherapy, radiotherapy, targeted therapy, and immunotherapy to control disease progression 3-5. Oxaliplatin, a key component of the standard FOLFOX regimen, is widely used for adjuvant and metastatic CRC treatment 6, 7. However, clinical studies reveal that approximately 30-40% of patients develop intrinsic or acquired resistance during therapy, markedly diminishing therapeutic efficacy and increasing recurrence risk 8-10. Moreover, oxaliplatin-induced neurotoxicity and other adverse effects often limit its prolonged use 11. Therefore, elucidating the molecular mechanisms underlying oxaliplatin resistance and developing novel intervention strategies are crucial for improving CRC treatment outcomes and patient survival.

The mechanisms driving oxaliplatin resistance in CRC are multifactorial, involving alterations in drug uptake and efflux, enhanced DNA repair, cell cycle dysregulation, inhibition of mitochondrial apoptosis, remodeling of the tumor microenvironment, and activation of cancer stemness pathways 12, 13. Although several resistance-associated genes and pathways, such as ABCC1, ERCC1, NF-κB, Notch, and Wnt/β-catenin, have been identified, most function merely as "accompanying phenomena" without offering viable therapeutic targets. With the advent of proteomic and multi-omic technologies, attention has shifted toward discovering deeper regulatory networks, particularly the role of small GTPase families in drug resistance. Small GTPases orchestrate cytoskeletal remodeling, cell migration, and endocytosis, processes crucial for tumor invasion, metastasis, and treatment resistance 14-16. Among them, the ARF (ADP-ribosylation factor) family is especially noteworthy 17, 18. However, the functions of ARF family members in CRC resistance remain largely unexplored. ARL4C (ARF-like 4C), a key member of the ARF family, has been implicated in promoting metastasis and a poor prognosis across various solid tumors through mechanisms that involve cytoskeletal remodeling, TGF-β pathway activation, and epithelial-mesenchymal transition (EMT) 19, 20.

Nevertheless, its expression pattern, pathological roles, and contribution to oxaliplatin resistance in CRC remain undefined. Our preliminary proteomic analysis revealed a significant upregulation of ARL4C in CRC clinical tissues exhibiting oxaliplatin treatment failure, suggesting its potential involvement in resistance mechanisms. Further analyses indicated that ARL4C simultaneously modulates the RAP1/PI3K-Akt/mTOR signaling loop and the RAC1/EMT axis, orchestrating both chemoresistance and metastatic potential. Moreover, we found that ARL4C sustains its pathogenic activity through a positive feedback loop by regulating deubiquitination processes and maintaining stable protein levels within the resistant microenvironment. These findings provide new insights into the complex molecular landscape of oxaliplatin resistance in CRC.

Building on these observations, the present study aimed to systematically dissect the role and mechanisms of ARL4C in mediating oxaliplatin resistance and distant metastasis in CRC, and to explore small-molecule inhibitors targeting ARL4C as potential therapeutic agents. Initially, we validated the overexpression and prognostic significance of ARL4C in resistant CRC tissues by analyzing clinical specimens and public datasets. We subsequently generated CRC cell lines with stable overexpression or knockdown of ARL4C, and evaluated their impact on oxaliplatin sensitivity, apoptosis, migration, and invasion through a series of *in vitro* and *in vivo* experiments. Proteomic profiling and co-immunoprecipitation assays were employed to elucidate ARL4C-associated signaling pathways and to investigate the regulation of its ubiquitination status. Furthermore, leveraging network pharmacology and molecular docking approaches, we identified β-Lapachone as a potential natural compound capable of targeting ARL4C. Structural modeling suggested that β-Lapachone can competitively bind to the LYS128 residue of ARL4C, potentially disrupting its interaction with DUBs, thereby promoting ARL4C degradation. This interaction can reverse oxaliplatin resistance, enhance antitumor efficacy, and mitigate renal toxicity. Given its integrative roles in signaling regulation, functional driving of tumor progression, and amenability to structural targeting, ARL4C represents a promising therapeutic vulnerability. Our findings pave the way for novel ARL4C-targeted interventions and lay a foundation for the development of personalized treatment strategies for chemoresistant CRC.

## Results

### Elevated ARL4C Expression in Oxaliplatin-Resistant CRC Correlates with Poor Prognosis

Oxaliplatin remains a cornerstone in the first-line chemotherapy regimens for CRC. It is widely utilized in adjuvant settings, advanced disease, and in neoadjuvant therapy for locally advanced cases to downstage tumors and improve resection rates 21, 22. However, oxaliplatin resistance is frequently encountered in clinical practice, significantly undermining treatment efficacy and exacerbating toxicity, ultimately leading to disease progression and poor patient outcomes 23, 24. To elucidate the mechanisms underlying oxaliplatin resistance, we stratified CRC patients undergoing neoadjuvant therapy into oxaliplatin-sensitive and -resistant groups based on preoperative imaging assessments using RECIST 1.1 criteria. Specifically, patients exhibiting a partial response (PR) or complete response (CR) were classified as oxaliplatin-sensitive, whereas those with stable disease (SD) or progressive disease (PD) were considered oxaliplatin-resistant. To validate this classification, we further evaluated the pathological response using tumor regression grade (TRG) according to AJCC/CAP guidelines. Patients with TRG 0-1 were defined as “responders (sensitive),” indicative of a favorable response and marked tumor regression, while those with TRG 2-3 were deemed “non-responders (resistant),” reflecting minimal or poor pathological response.

**Figure 1:**
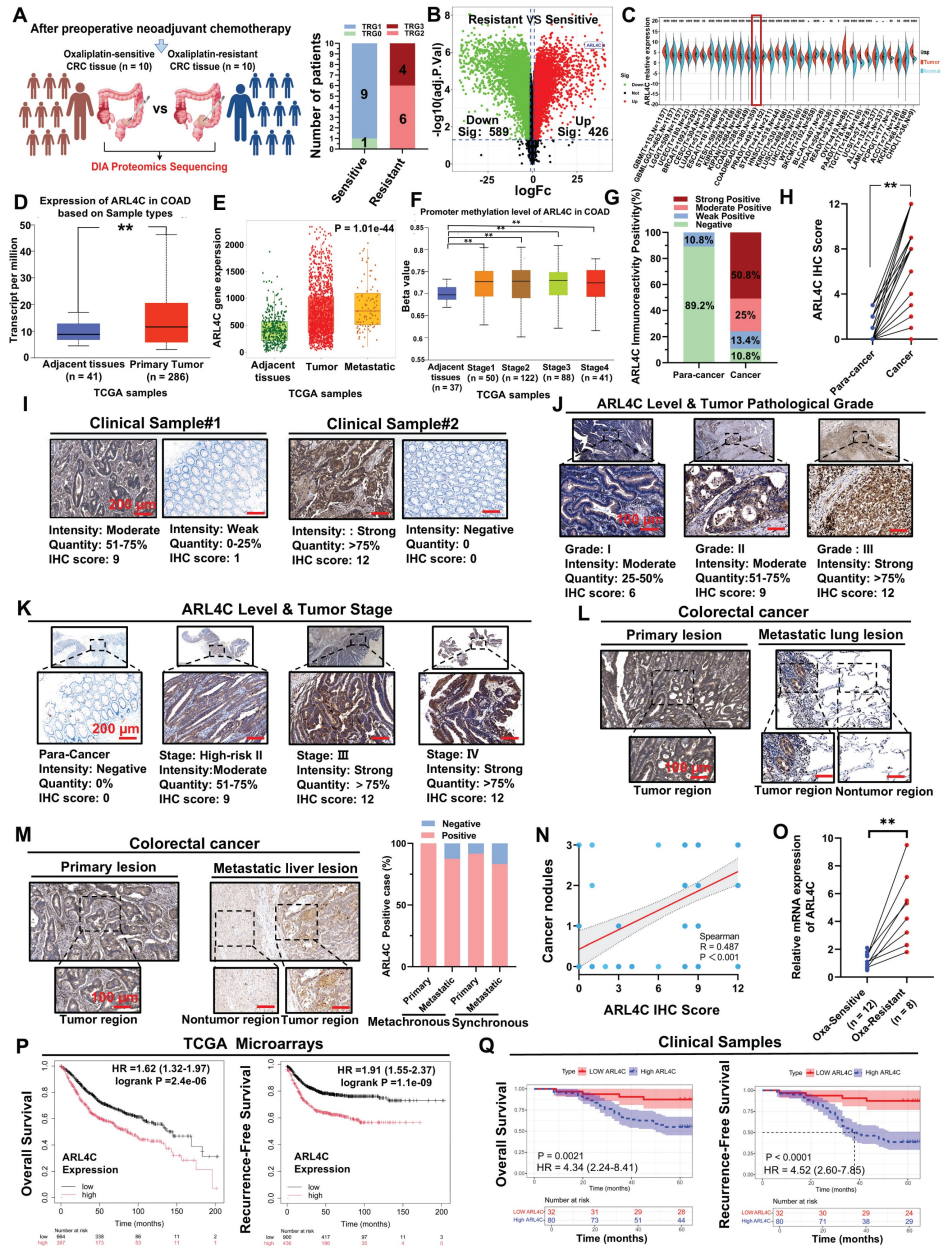
** ARL4C is overexpressed in oxaliplatin-resistant colorectal cancer tissues and correlates with poor prognosis. (A)** Proteomic sequencing (DIA) was conducted in enrolled patients, comprising 10 oxaliplatin-resistant and 10 oxaliplatin-sensitive cases following neoadjuvant therapy and preoperative imaging evaluation. **(B)** Volcano plot showing differentially expressed genes (DEGs) in oxaliplatin-resistant and sensitive patients (426 upregulated, 589 downregulated). **(C)** Pan-cancer analysis of ARL4C gene expression using Sangerbox. **(D)** Expression of ARL4C in colon cancer and adjacent tissues from the TCGA dataset using the UALCAN database. **(E)** ARL4C expression in colorectal cancer, metastatic lesions, and adjacent tissues analyzed through TNMplot. **(F)** ARL4C expression changes across different stages of colorectal cancer using the UALCAN database. **(G)** ARL4C immunohistochemistry positivity rates in cancer and para-cancer tissues from 112 clinical samples treated with oxaliplatin. **(H)** Paired comparison of ARL4C expression in cancer and para-cancer tissues in oxaliplatin-treated clinical samples. **(I)** ARL4C immunohistochemistry results in cancer and para-cancer tissues from oxaliplatin-treated samples. **(J)** Immunohistochemistry results showing ARL4C expression with increasing pathological grading in oxaliplatin-treated colorectal cancer samples. **(K)** ARL4C immunohistochemistry results with clinical staging in oxaliplatin-treated samples. **(L)** ARL4C immunohistochemistry results in primary and lung metastatic lesions from oxaliplatin-treated samples. **(M)** ARL4C immunohistochemistry results in primary and liver metastatic lesions from oxaliplatin-treated samples. The right panel shows the percentage of ARL4C-positive cases in primary and metastatic lesions from synchronous and metachronous liver metastases. **(N)** Correlation of ARL4C expression with the number of cancer nodules in colorectal cancer patients (r = 0.487, p < 0.001). **(O)** Paired comparison of ARL4C expression in tissues before and after oxaliplatin resistance in clinical samples. **(P)** Correlation between ARL4C expression levels and prognosis in colorectal cancer patients in the TCGA dataset. **(Q)** Correlation between ARL4C expression levels and prognosis in 112 clinical oxaliplatin-treated samples. (* p < 0.05, ** p < 0.01, *** p < 0.001, **** p < 0.0001, ns. p > 0.05).

TRG-based classification demonstrated a consistent trend with the imaging-based stratification, supporting the robustness of our grouping strategy (Figure 1A). Subsequently, DIA-based proteomic profiling was performed on tumor specimens to identify molecular features associated with oxaliplatin resistance. Comparative analysis identified 426 proteins significantly upregulated in the resistant group (Figure 1B). Among the top 10 differentially expressed proteins, ARL4C, a member of the ARF family of small GTPases, captured our interest. Small GTPases, as critical molecular switches, orchestrate a wide range of cellular processes, including signal transduction, membrane trafficking, cell polarity, and cytoskeletal remodeling. Recent insights have highlighted their pivotal roles in tumor proliferation, metastasis, and therapy resistance, positioning them as emerging targets in oncology 25, 26. ARL4C is known to be upregulated across multiple tumor types and promotes proliferation and migration via pathways, such as the Wnt/β-catenin pathway 27. However, its involvement in oxaliplatin resistance in CRC remains unexplored.

Pan-cancer analyses revealed that ARL4C is significantly overexpressed in 23 tumor types, including CRC, compared with normal tissues (Figure 1C). Further analysis using the TNMplot and UALCAN databases confirmed elevated ARL4C expression in colon cancer tissues compared to adjacent tissues (Figure 1D, Figure S1A). This expression was even higher in metastatic lesions than in primary tumors and adjacent tissues (Figure 1E). Moreover, ARL4C levels were markedly higher in mucinous adenocarcinomas, characterized by a higher degree of malignancy, compared with conventional adenocarcinomas and adjacent tissues (Figure S1B). Notably, ARL4C expression progressively increased with advancing tumor stage and lymph node involvement (Figure 1F, Figure S1C-D). TCGA analysis revealed a positive correlation between ARL4C expression and tumor mutational burden (TMB) (Figure S1E). Although elevated TMB is generally associated with improved immunotherapy response, we observed that most pMMR-type CRCs (typically immunotherapy-refractory) exhibited high ARL4C expression (Figure S1F), suggesting a complex interplay between ARL4C, genomic instability, and immune responsiveness.

To further validate these findings, we collected clinical specimens and data from 112 patients with CRC who were treated with oxaliplatin (Table S1). Patients with histologically confirmed colorectal adenocarcinoma who underwent curative-intent resection were included if they met one of the following criteria: high-risk stage II disease (defined as T4 stage, poor differentiation, lymphovascular or perineural invasion, bowel obstruction or perforation, or <12 lymph nodes examined), stage III disease, or stage IV disease with resectable or treated metastases eligible for adjuvant chemotherapy. All included patients received oxaliplatin-based adjuvant chemotherapy. Exclusion criteria included stage I or low-risk stage II disease, absence of surgical treatment, incomplete clinical data, or contraindications to chemotherapy. Consistent with bioinformatics analyses, ARL4C expression was markedly higher in cancer tissues than in para-cancer tissues (Figure 1G-I). ARL4C levels correlated positively with pathological grade and clinical stage (Figure 1J-K, Table S2). Higher carcinoembryonic antigen (CEA) levels, a commonly used biomarker in CRC, were also associated with elevated ARL4C expression (Figure S1G). Significantly, in patients with liver and lung metastases, ARL4C expression was elevated in both primary and metastatic lesions relative to adjacent normal tissues (Figure 1L-M). Furthermore, ARL4C levels showed a positive correlation with cancer nodules (R = 0.487) and were associated with increased perineural invasion (59.8% vs. 4.5%) (Figure 1N, Figure S1H).

Although immunohistochemical analysis revealed no significant difference in ARL4C expression between primary and metastatic lesions, our analysis of the GSE231559 single-cell RNA sequencing dataset uncovered a marked increase in ARL4C expression in metastatic sites compared to primary tumors (Figure S1I-K). Leveraging the GDSC database, we identified a negative correlation between ARL4C expression and oxaliplatin sensitivity (Figure S1L). Paired comparisons further confirmed that ARL4C expression was significantly higher in oxaliplatin-resistant tumors compared to those that were sensitive (Figure 1O).

These findings strongly implicated ARL4C in the acquisition of oxaliplatin resistance in CRC. Univariate Cox regression analyses similarly highlighted ARL4C expression, Ki-67 proliferation index, TNM stage, and MMR status as substantial correlates of overall survival (OS), as well as ARL4C expression, tumor pathological grade, TNM stage, and MMR status as significant correlates of recurrence-free survival (RFS) (Table S3). Multivariate Cox regression analyses demonstrated that ARL4C immunohistochemistry (IHC) scores, TNM stage, and MMR status were independent prognostic factors for both OS and RFS (Table S4).

Further survival analyses using the TCGA cohort and the TIMER2.0 platform revealed that high ARL4C expression was significantly associated with worse OS, progression-free survival (PFS), and post-progression survival (PPS) (Figure S1M-O). In colon cancer-specific subgroup analyses, ARL4C consistently emerged as a risk factor for OS, PFS, and disease-specific survival (DSS) (Figure S1P-R). Pan-cancer analyses further underscored the prognostic relevance of ARL4C across multiple malignancies (Figure S1S). Predictive models integrating ARL4C expression accurately forecasted 1-year, 3-year, and 5-year RFS and OS in CRC patients (Figure 1P). In our clinical cohort, patients with high ARL4C expression exhibited significantly shorter RFS and OS compared to those with low expression (Figure 1Q).

In summary, through comprehensive bioinformatic and clinical analyses, we demonstrate that ARL4C is closely associated with CRC progression, oxaliplatin resistance, and poor prognosis. Nevertheless, the molecular mechanisms underlying ARL4C-mediated chemoresistance warrant further investigation.

### ARL4C Mediates Oxaliplatin Resistance in CRC Cells by Regulating Apoptosis and EMT

**Figure 2:**
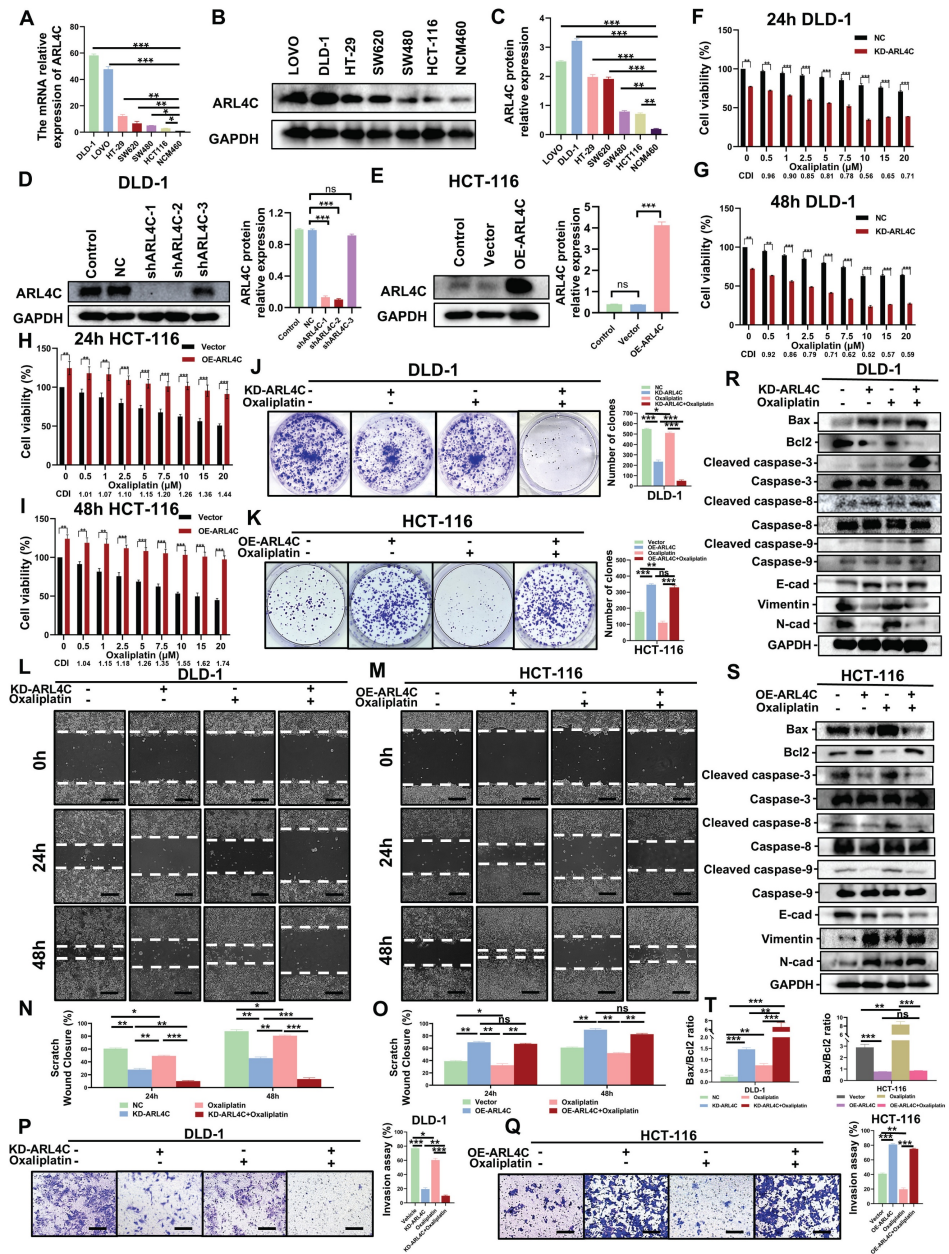
** ARL4C promotes oxaliplatin resistance in colorectal cancer cells by regulating apoptosis and EMT. (A)** mRNA expression of ARL4C in six colorectal cancer cell lines (DLD-1, LOVO, HT-29, SW620, SW480, HCT-116) and NCM460 normal colon epithelial cells. **(B-C)** Protein expression levels of ARL4C in these cell lines. **(D)** Verification of ARL4C knockdown efficiency in DLD-1 cells. **(E)** Verification of ARL4C overexpression efficiency in HCT-116 cells. **(F-G)** Cell viability changes in DLD-1-KD-ARL4C cells with oxaliplatin treatment at 24h and 48h. **(H-I)** Cell viability changes in HCT-116-OE-ARL4C cells with oxaliplatin treatment at 24h and 48h. **(J-K)** Clonogenic assays to examine the effects of ARL4C knockdown/overexpression and oxaliplatin treatment on colorectal cancer cell proliferation. **(L-O)** Wound healing assays to assess the impact of ARL4C knockdown/overexpression and oxaliplatin treatment on colorectal cancer cell migration. **(P-Q)** Transwell assays to evaluate the effects of ARL4C knockdown/overexpression and oxaliplatin treatment on colorectal cancer cell invasion. **(R-T)** Western blot analysis of apoptosis and EMT-related proteins in colorectal cancer cells with ARL4C knockdown/overexpression and oxaliplatin treatment. (* p < 0.05, ** p < 0.01, *** p < 0.001, ns. p > 0.05).

To further investigate the role of ARL4C in CRC progression and oxaliplatin resistance, we first evaluated ARL4C expression at both mRNA and protein levels across a panel of CRC cell lines (DLD-1, LOVO, HT-29, SW620, SW480, HCT-116) and a normal colon epithelial cell line (NCM460). Our results revealed that DLD-1 cells exhibited the highest ARL4C expression, whereas HCT-116 cells showed the lowest expression at both RNA and protein levels (Figure 2A-C). Intriguingly, a dose-dependent cell viability determination assay (Figure S2A) exhibited a negative correlation between ARL4C expression and oxaliplatin sensitivity in six CRC cell lines at 72 h after oxaliplatin treatment (Figure S2A). This observation was consistent with our findings in clinical specimens, where the DLD-1 cell line was the most oxaliplatin-resistant and the HCT-116 cell line was the most sensitive. Based on these findings, we chose to knock down ARL4C in the DLD-1 cell line, which has the highest expression of ARL4C and is drug resistant, and overexpress it in the HCT-116 cell line, which is sensitive and has a low expression of ARL4C, for further functional analysis. We confirmed the validity of the knockdown and overexpression by qRT-PCR and Western blotting. (Figure 2D-E). Interestingly, ARL4C depletion significantly sensitized DLD-1 cells to oxaliplatin (Figure 2F-G), while ARL4C overexpression rendered HCT-116 cells more resistant to oxaliplatin (Figure 2H-I), further validating our hypothesis that ARL4C plays a pivotal role in CRC oxaliplatin resistance and may represent a promising therapeutic target to overcome drug resistance.

To substantiate these findings, we conducted clonogenic assays to assess the proliferative capacity of CRC cells following ARL4C modulation with or without oxaliplatin treatment. ARL4C knockdown moderately suppressed DLD-1 proliferation. Notably, low-dose oxaliplatin alone exerted minimal cytotoxic effects; however, combined ARL4C silencing and oxaliplatin treatment markedly enhanced growth inhibition compared to either intervention alone (Figure 2J).

Similar results were observed in HCT-116 cells, where ARL4C overexpression promoted resistance to oxaliplatin (Figure 2K), a finding further corroborated by EdU assays (Figure S2B). Next, we performed wound healing and Transwell assays to evaluate the effects of ARL4C on cell migration and invasion upon oxaliplatin treatment. ARL4C knockdown in DLD-1 cells significantly suppressed migration and invasion, especially when combined with oxaliplatin, while ARL4C overexpression in HCT-116 cells enhanced their migratory and invasive abilities (Figure 2L-Q). These results were further validated by ARL4C knockdown in LOVO cells, which showed consistent trends (Figure S3A-E).

Given that oxaliplatin primarily exerts antitumor effects through apoptosis induction, we next explored ARL4C's impact on apoptosis. Calcein-AM/PI assays demonstrated that ARL4C depletion enhanced apoptosis in DLD-1 cells, which was significantly amplified when combined with oxaliplatin treatment (Figures S2C-D). In contrast, ARL4C overexpression counteracted oxaliplatin-induced apoptosis in HCT-116 cells. We subsequently analyzed the expression of apoptosis-related proteins under these conditions. ARL4C knockdown or oxaliplatin treatment alone led to increased levels of pro-apoptotic proteins (BAX, Cleaved caspase-3/8/9), while combined treatment further elevated their expression (Figure 2R). Conversely, the anti-apoptotic protein Bcl-2 was downregulated, with the greatest reduction observed upon combined ARL4C knockdown and oxaliplatin exposure. Notably, ARL4C overexpression in HCT-116 cells suppressed these apoptotic responses (Figure 2S). Collectively, these data suggested that ARL4C inhibition synergizes with oxaliplatin to enhance apoptosis in CRC cells.

Since EMT plays a crucial role in tumor metastasis, invasion, and drug resistance, we assessed the expression of EMT-related proteins. ARL4C knockdown increased epithelial marker E-cadherin while decreasing mesenchymal markers N-cadherin and Vimentin, indicative of EMT suppression and tumor-suppressive effects (Figure 2R). Interestingly, low-dose oxaliplatin treatment alone did not significantly alter EMT markers in DLD-1 cells. However, in HCT-116 cells, it promoted EMT, as evidenced by the downregulation of E-cadherin and upregulation of N-cadherin and Vimentin. These observations suggested that EMT activation may serve as a compensatory survival mechanism against oxaliplatin cytotoxicity. Moreover, in HCT-116 cells, simultaneous ARL4C overexpression and oxaliplatin exposure led to more pronounced EMT activation compared to either intervention alone, whereas combined ARL4C knockdown and oxaliplatin treatment effectively inhibited EMT (Figure 2R-S, Figure S2E-F). These findings highlighted ARL4C as a critical mediator of oxaliplatin resistance via EMT regulation. We further validated the results in LOVO cells with ARL4C knockdown (Figure S3F-G). It is worth noting that DLD-1, HCT116, and LOVO are all classified as MSI (microsatellite instability) cell lines, which eliminates the influence of key biological confounding factors in this study.

### ARL4C Promotes Tumor Progression, Metastasis, and Mediates Oxaliplatin Resistance in CRC *In Vivo*

**Figure 3:**
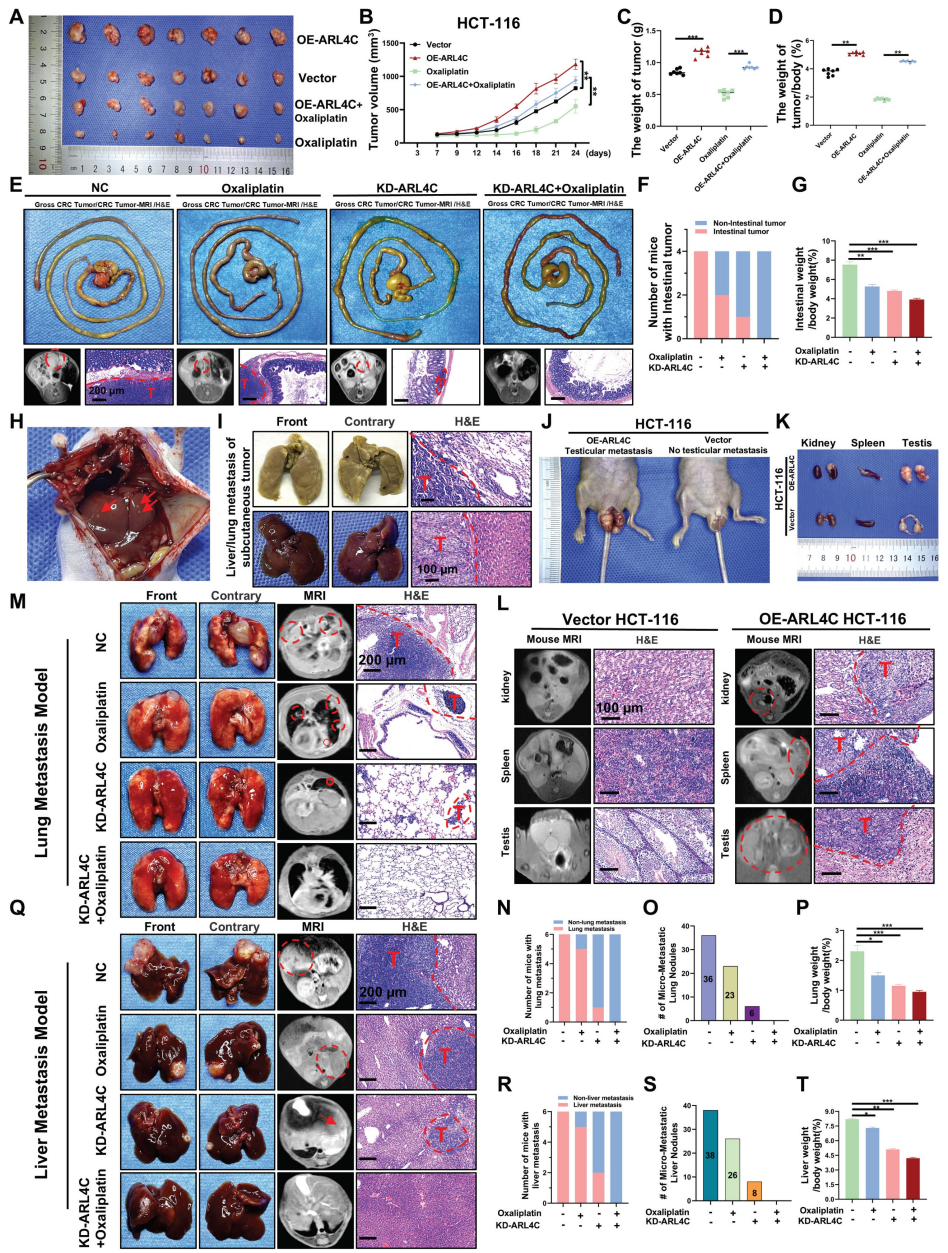
** ARL4C enhances tumor progression, metastasis, and contributes to oxaliplatin resistance in colorectal cancer in vivo. (A)** Representative images of xenografts treated with ARL4C-overexpressing HCT-116 or oxaliplatin (10 mg/kg). **(B)** Tumor volume of xenografts. **(C)** Tumor weight of xenografts. **(D)** Ratio of tumor weight to body weight in xenografts. **(E)** Impact of ARL4C knockdown and oxaliplatin treatment on DLD-1 orthotopic tumor growth (images, MRI scans, and H&E staining). **(F)** Number of mice with intestinal tumors. **(G)** Ratio of intestinal weight to body weight in the orthotopic xenograft model. **(H)** Liver metastasis observed in mice with subcutaneous tumors from HCT-116 ARL4C overexpression. **(I)** Representative images and H&E staining of lung and liver metastasis in HCT-116 ARL4C overexpression xenografts. **(J)** Representative images of testicular metastasis in HCT-116 ARL4C overexpression xenografts. **(K-L)** Representative images, MRI scans, and H&E staining of kidney, spleen, and testicular metastasis in HCT-116 ARL4C overexpression xenografts. **(M)** Representative images of lung metastasis in mice treated with ARL4C knockdown and oxaliplatin groups. **(N)** Number of mice with or without lung metastasis in each group. **(O)** MRI detection of micro-metastatic lung nodules in each group. **(P)** Lung weight to body weight ratio for each group. **(Q-T)** Impact of ARL4C knockdown and oxaliplatin treatment on liver metastasis in DLD-1 cells. (* p < 0.05, ** p < 0.01, *** p < 0.001, ns. p > 0.05).

We established a subcutaneous xenograft model using HCT116 cells with stable ARL4C overexpression in Balb/c nude mice to elucidate further the role of ARL4C in CRC progression and oxaliplatin resistance. We evaluated tumor growth with or without oxaliplatin treatment. Notably, ARL4C overexpression markedly accelerated subcutaneous tumor growth and completely abrogated the antitumor effects of oxaliplatin (Figure 3A-D). To standardize tumor burden assessment across individual mice, we employed the tumor weight-to-body weight ratio, thereby minimizing the confounding effects of systemic toxicity such as weight loss. The results consistently confirmed ARL4C's role in promoting tumor proliferation (Figure 3D). Immunohistochemical analysis further revealed that ARL4C overexpression enhanced Ki67 expression, while suppressing cleaved caspase-3 and E-cadherin (Figure S4A). Notably, ARL4C overexpression completely reversed oxaliplatin-induced tumor suppression, mirroring our *in vitro* findings and underscoring ARL4C as a potential driver of oxaliplatin resistance.

In preclinical cancer research, model selection depends on the specific biological question being addressed. Subcutaneous models are easy to establish and ideal for evaluating tumor growth and drug efficacy, but they lack the native microenvironment, which limits studies on invasion, metastasis, and immune response. Compared to conventional subcutaneous models, orthotopic CRC models mimic tumor behavior in the native site, preserving interactions with the mucosa, immune cells, and microbiota, and are better suited for studying tumor-host interactions, progression, and metastatic spread 28. To validate the effects of ARL4C in a more physiologically relevant setting, we generated an orthotopic CRC model using DLD-1 cells with ARL4C knockdown (Figure 3E). Remarkably, combined ARL4C knockdown and oxaliplatin treatment completely suppressed tumor growth (Figure 3F-G), suggesting that targeting ARL4C could significantly enhance oxaliplatin efficacy in CRC patients.

Strikingly, mice bearing ARL4C-overexpressing tumors developed extensive spontaneous metastases, with macroscopic nodules detected in the liver and lungs (Figure 3H, I). In several cases, metastases were also observed in the kidneys, spleen, and testes (Figure 3J-L). We further validated this observation by establishing a lung metastasis model via tail-vein injection using HCT-116 and HCT-116-OE-ARL4C cells. Compared with the HCT-116 group, mice injected with HCT-116-OE-ARL4C cells developed a higher number of lung metastases characterized by more metastatic nodules and larger tumor burdens (Figure S4B). These findings highlighted a previously unappreciated role of ARL4C in promoting metastasis in CRC.

Since spontaneous metastases were observed with ARL4C overexpression (Figure 3H-L), we investigated its role in CRC metastasis. Using a DLD-1 ARL4C-knockdown lung metastasis model, we found that ARL4C depletion alone exerted stronger metastasis-suppressing effects than oxaliplatin monotherapy (Figure 3M), evidenced by a dramatic reduction in pulmonary metastatic nodules (Figure 3N-P). Notably, the combination of ARL4C knockdown with oxaliplatin significantly abolished lung metastasis, suggesting a potent synergistic effect. Considering the clinical relevance of liver metastasis, which affects over 50% of advanced CRC patients, we established a DLD-1 ARL4C-knockdown liver metastasis model (Figure 3Q). Consistent with the lung metastasis results, ARL4C depletion markedly reduced hepatic metastatic burden, and its combination with oxaliplatin nearly eradicated liver metastasis formation (Figure 3R-T). Collectively, these data demonstrated that ARL4C plays a crucial role in CRC proliferation, metastasis, and chemoresistance. Thus, therapeutically targeting ARL4C, particularly in combination with oxaliplatin, represents a promising strategy to overcome metastasis and drug resistance in CRC.

### CRC Cells Promote Oxaliplatin Resistance via ARL4C Ubiquitination Modulated by the ARL4C/RAP1/PI3K-Akt-mTOR Signaling Loop

**Figure 4:**
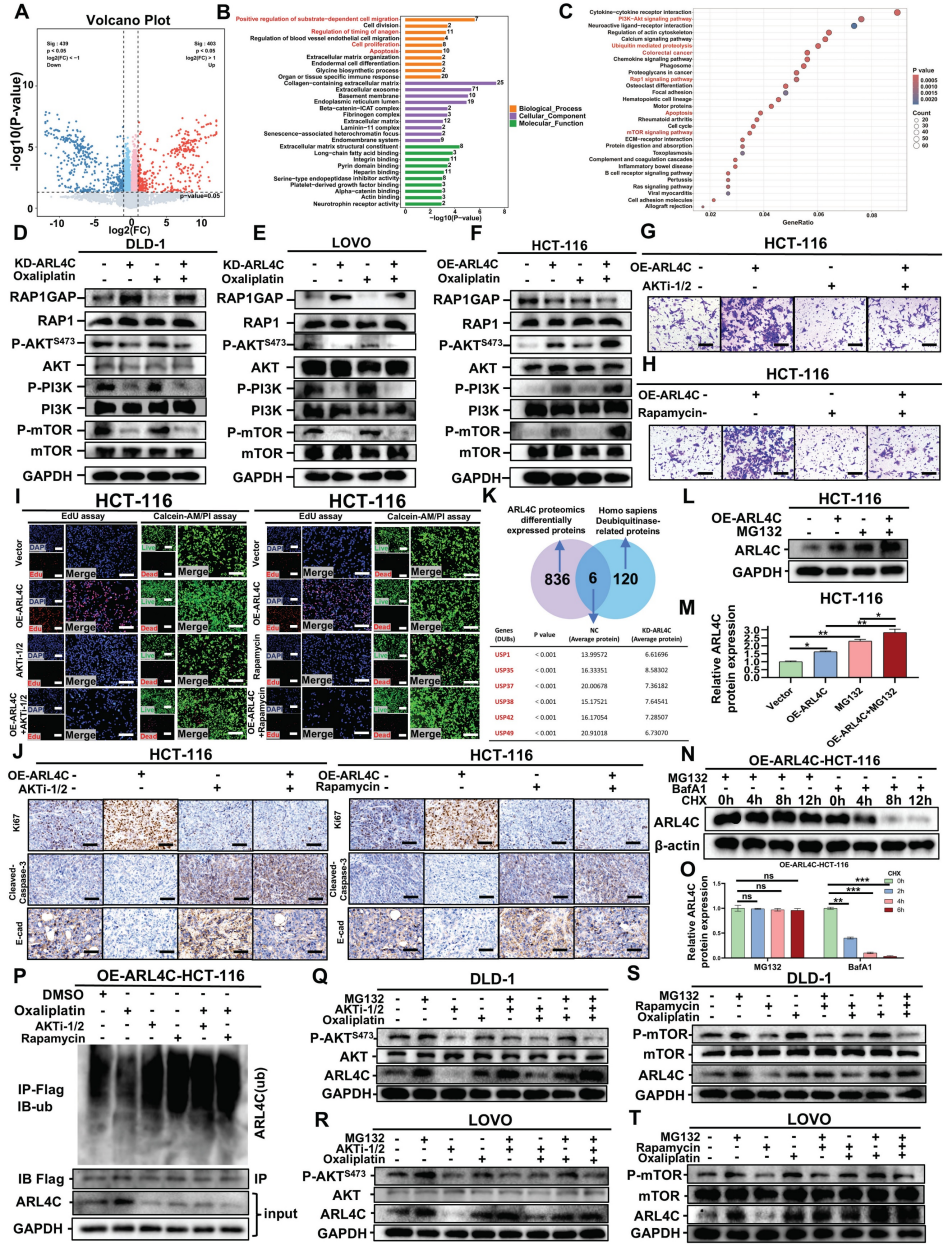
** Colorectal cancer cells regulate ARL4C ubiquitination via the ARL4C/RAP1/PI3K-Akt-mTOR signaling loop to mediate oxaliplatin resistance. (A)** Volcano plot of differentially expressed genes (403 upregulated, 439 downregulated) following ARL4C knockdown detected by proteomics sequencing. **(B)** GO enrichment analysis of differentially expressed genes in proteomics sequencing. **(C)** KEGG enrichment analysis of differentially expressed genes in proteomics sequencing. **(D-F)** Western blot analysis of pathway-related proteins in colorectal cancer cells following ARL4C knockdown/overexpression and oxaliplatin treatment.** (G)** Transwell assays to assess the effect of ARL4C overexpression and AKTi-1/2 treatment on colorectal cancer cell invasion. **(H)** Transwell assays to examine the impact of ARL4C overexpression and rapamycin treatment on colorectal cancer cell invasion. **(I)** EdU assays and Live/dead cell staining to evaluate the effects of ARL4C overexpression and AKTi-1/2 or rapamycin treatment on colorectal cancer cell proliferation. **(J)** Immunohistochemistry to detect changes in proliferation, apoptosis, and EMT-related protein expression in subcutaneous tumors of HCT-116 cells treated with ARL4C overexpression and AKTi-1/2 or rapamycin. **(K)** Venn diagram showing the intersection of differentially expressed proteins from proteomics sequencing and ubiquitination-related proteins, identifying six deubiquitinating enzymes. **(L-M)** Western blot analysis of ARL4C protein expression following ARL4C overexpression and MG132 treatment. **(N-O)** Western blot analysis of ARL4C protein expression following ARL4C overexpression and MG132/BafA1/CHX treatment. **(P)** CoIP assays to investigate changes in ARL4C ubiquitination levels following ARL4C overexpression and AKTi-1/2 or rapamycin treatment. **(Q-R)** Western blot analysis of ARL4C and P-AKT^S473^ expression following treatment with MG132, AKTi-1/2, or oxaliplatin. **(S-T)** Western blot analysis of ARL4C and P-mTOR expression following treatment with MG132, rapamycin, or oxaliplatin. (* p < 0.05, ** p < 0.01, *** p < 0.001, ns. p > 0.05).

We examined the mechanisms by which ARL4C contributes to oxaliplatin resistance in CRC by performing proteomic profiling to assess global protein expression changes following ARL4C knockdown. A total of 403 and 439 proteins were significantly upregulated or downregulated, respectively (Figure 4A). Gene Ontology (GO) enrichment analysis revealed that these differentially expressed proteins were mainly involved in positive regulation of substrate-dependent cell migration, regulation of anagen timing, cell proliferation, and apoptosis (Figure 4B). Kyoto Encyclopedia of Genes and Genomes (KEGG) pathway analysis indicated enrichment in the PI3K-Akt signaling pathway, ubiquitin-mediated proteolysis, RAP1 and mTOR signaling pathways, and apoptosis (Figure 4C). Previous studies have demonstrated that RAP1, PI3K-Akt, and mTOR pathways form an integrated signaling network crucial for regulating cell proliferation, survival, migration, and metabolism, all of which are key factors in tumor progression and drug resistance 29, 30. Activated RAP1 recruits and activates PI3K, subsequently initiating the PI3K-Akt pathway. mTOR acts as a downstream effector, executing the biological outcomes of RAP1-PI3K-Akt signaling. Collectively, these pathways establish the RAP1/PI3K-Akt/mTOR axis, intimately involved in EMT, metabolic reprogramming, and therapy resistance.

Next, we assessed the expression of key proteins within the RAP1, PI3K-Akt, and mTOR pathways. ARL4C knockdown significantly inhibited the RAP1/PI3K-Akt/mTOR axis in DLD-1 and LOVO cells (Figure 4D-E, Figure S5A-B), whereas ARL4C overexpression activated this axis in HCT-116 cells (Figure 4F, Figure S5C), confirming a positive regulatory relationship. Dual immunofluorescence further validated this positive correlation (Figure S5D-E). Interestingly, exposure to oxaliplatin significantly activated the RAP1 pathway and moderately activated the PI3K-Akt and mTOR pathways (Figure 4D-F, Figure S5A-C), suggesting a compensatory activation of the RAP1/PI3K-Akt/mTOR axis in response to chemotherapy-induced stress. Notably, ARL4C knockdown abolished this compensatory activation, whereas ARL4C overexpression markedly enhanced it, positioning ARL4C as a key mediator of oxaliplatin resistance via modulation of this signaling axis.

To further validate the role of the RAP1/PI3K-Akt/mTOR pathway in ARL4C-mediated resistance, we treated cells with the AKT inhibitor (AKT i-1/2) and mTOR inhibitor (rapamycin). Transwell assays demonstrated that both inhibitors partially reversed the ARL4C-induced invasive phenotype in HCT-116 cells (Figures 4G-H and S5F-G). More strikingly, EdU and Calcein-AM/PI assays revealed that AKT i-1/2 and rapamycin abrogated ARL4C-induced proliferation and survival advantages (Figure 4I, Figure S5H-K), suggesting that ARL4C predominantly regulates proliferation and apoptosis through the RAP1/PI3K-Akt/mTOR axis, while invasion may involve additional parallel pathways. Consistent findings were observed *in vivo* using a cell-derived xenograft (CDX) model, where both inhibitors fully reversed the pro-tumorigenic effects of ARL4C on proliferation and apoptosis, and partially suppressed EMT (Figure 4J, Figure S5L-Q). TCGA database analysis further revealed positive correlations between ARL4C expression and PI3K-Akt/mTOR signaling, ECM degradation, and EMT markers (Figure S5R-T).

Proteomic analysis also highlighted a close association between ARL4C and the ubiquitin-proteasome pathway, identifying six differentially expressed deubiquitinases (DUBs) (USP1, USP35, USP37, USP38, USP42, USP49) (Figure 4K). ARL4C knockdown led to a notable downregulation of these DUBs. The proteasome inhibitor MG132 treatment could increase ARL4C expression and significantly inhibit its degradation (Figure 4L-O), indicating that ARL4C degradation is proteasome-dependent. Further, immunoprecipitation assays showed that oxaliplatin treatment decreased ARL4C ubiquitination levels compared to controls, suggesting that tumor cells may attenuate ARL4C degradation to resist oxaliplatin cytotoxicity (Figure 4P). Notably, AKT i-1/2 and rapamycin restored ARL4C ubiquitination levels, even under oxaliplatin treatment, indicating that the RAP1/PI3K-Akt/mTOR axis regulates ARL4C stability. Previous reports have shown that mTOR activation can upregulate DUBs by enhancing translational and transcriptional programs through downstream effectors such as S6K and 4EBP1 or through transcription factors like HIF-1α, MYC, and NF-Κb 31, consistent with our findings (Figure 4K). TCGA analysis confirmed positive correlations between ARL4C and DUBs expression (Figure S6A-F). Molecular docking further predicted direct interactions between ARL4C and several DUBs (Figure S6G-K, Table S5). Thus, we propose that ARL4C activation of the RAP1/PI3K-Akt/mTOR axis upregulates DUB expression, thereby reducing its own ubiquitination and degradation, a mechanism particularly enhanced under oxaliplatin treatment.

Pharmacological inhibition of AKT (AKT i-1/2) significantly decreased P-AKT(S473) and ARL4C levels (Figure 4Q-R), supporting a positive feedback loop between ARL4C and the PI3K-Akt pathway. Oxaliplatin treatment increased both P-AKT and ARL4C levels, further supporting this feedback mechanism. Importantly, MG132 treatment abolished the AKT i-1/2-induced reduction of ARL4C, indicating that the regulatory effect is proteasome-dependent. Similarly, rapamycin treatment suppressed both P-mTOR and ARL4C levels (Figure 4S-T), and MG132 abolished this suppression. These findings underscore the proteasome dependency of ARL4C regulation via mTOR signaling. Additionally, combined treatments with MG132 and oxaliplatin did not show additive effects compared to single treatments (Figure 4Q-T, Figure S7A-H), suggesting that oxaliplatin stabilizes ARL4C primarily by inhibiting its ubiquitin-mediated degradation. Collectively, these data established that CRC cells promote oxaliplatin resistance by modulating ARL4C ubiquitination through the ARL4C/RAP1/PI3K-Akt-mTOR signaling loop.

### Upregulation of USP38 by ARL4C Reduces Its Ubiquitination Level to Enhance RAP1/PI3K-Akt/mTOR Signaling Loop

**Figure 5:**
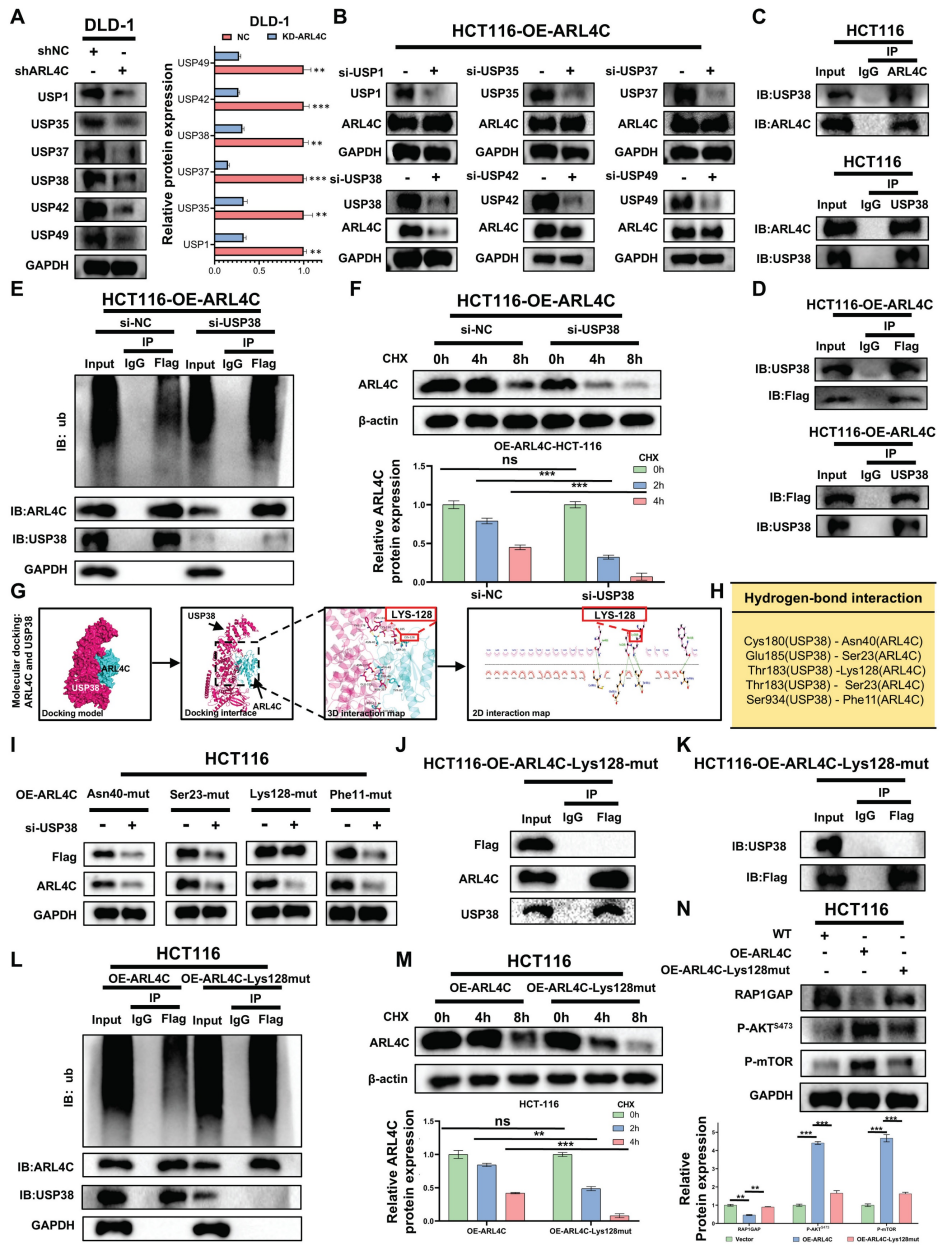
** ARL4C-mediated upregulation of USP38 reduces its own ubiquitination, enhancing the RAP1/PI3K-Akt/mTOR signaling loop. (A)** Western Blot analysis of protein changes of ARL4C following knockdown of six deubiquitinases. **(B)** Western Blot analysis of protein changes of ARL4C following knockdown of six deubiquitinases. **(C-D)** Co-IP experiments detecting the interaction between ARL4C and USP38. **(E)** Western Blot analysis of the effect of USP38 knockdown on ARL4C ubiquitination levels. **(F)** Western Blot analysis of the effect of USP38 knockdown on ARL4C degradation rate under CHX treatment. **(G-H)** 2D and 3D molecular docking of ARL4C and USP38 and potential interaction sites. **(I)** Western Blot analysis of ARL4C levels under four mutation sites and USP38 knockdown conditions. **(J-K)** Co-IP analysis of the interaction between ARL4C and USP38 in the case of Lys128 mutation in ARL4C. **(L)** Western Blot analysis of the effect of ARL4C-Lys128-mut on ARL4C ubiquitination levels. **(M)** Western Blot analysis of the effect of ARL4C-Lys128-mut on ARL4C degradation rate under CHX treatment. **(N)** Western Blot analysis of the effect of ARL4C-Lys128-mut on the RAP1/PI3K-Akt-mTOR signalin loop. (* p < 0.05, ** p < 0.01, *** p < 0.001, ns. p > 0.05).

In our previous study, we identified six DUBs (USP1, USP35, USP37, USP38, USP42, USP49) that were positively correlated with ARL4C expression and had the potential for interaction with ARL4C, as indicated by proteomics analysis (Figure 4K). We also discovered that the ubiquitination and degradation of ARL4C are closely linked to the regulation of the RAP1/PI3K-Akt/mTOR signaling pathway. We further explored the roles of the six DUBs in ARL4C signaling regulation by downregulating their expression in the ARL4C-knockdown DLD-1 cell line (Figure 5A). Interestingly, in the ARL4C-overexpressing HCT116 cell line, when we knocked down each of the six DUBs, only the knockdown of USP38 resulted in a decrease in ARL4C protein levels (Figure 5B), indicating that USP38 plays a role in regulating ARL4C protein expression.

We further investigated the relationship between USP38 and ARL4C by performing CoIP experiments, which revealed that both endogenous and exogenous ARL4C interact with USP38 (Figure 5C-D). Additionally, knockdown of USP38 led to a significant increase in ARL4C's ubiquitination level (Figure 5E) and accelerated its degradation (Figure 5F). Through molecular docking, we identified four potential interaction sites on ARL4C (Asn40, Ser23, Lys128, Phe11) that may bind to USP38 (Figure 5G-H). We then constructed ARL4C mutants for each of these four sites and found that only the mutation at Lys128 abolished USP38's regulatory effect on ARL4C (Figure 5I). Furthermore, the Lys128 mutation disrupted the interaction between ARL4C and USP38 (Figure 5J-K). Along with the Lys128 mutation, ARL4C's ubiquitination level significantly increased, and its degradation rate accelerated (Figure 5L-M). Interestingly, the mutation at Lys128 reversed the effect of ARL4C overexpression on the regulation of the RAP1/PI3K-Akt/mTOR signaling pathway (Figure 5N). In summary, ARL4C interacts with USP38 at the Lys128 site to stabilize its protein level, thereby activating the RAP1/PI3K-Akt/mTOR signaling pathway.

### ARL4C Promotes Oxaliplatin Resistance in CRC by Regulating RAC1/Arp2/3 Signaling Axis to Induce EMT

**Figure 6:**
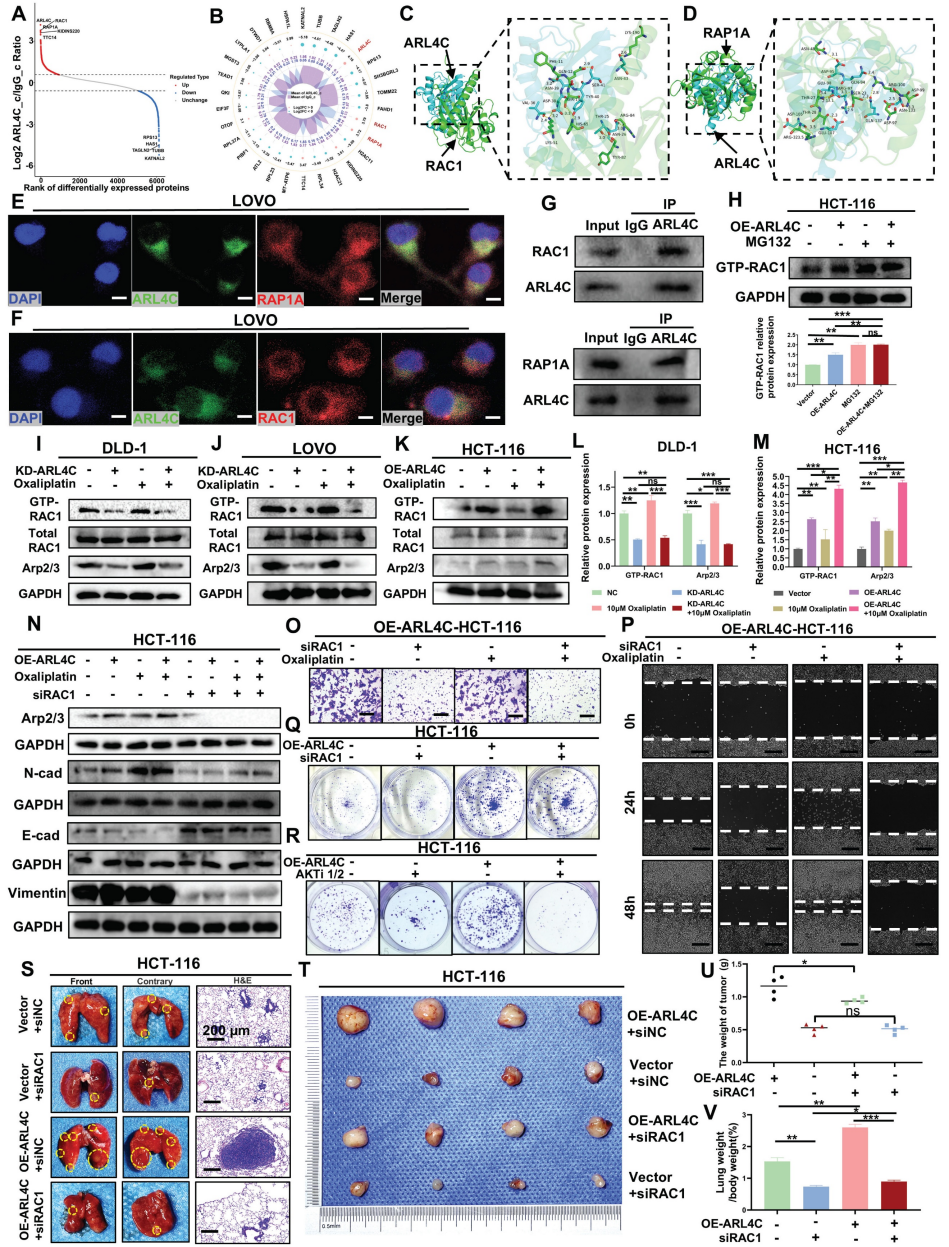
** Colorectal cancer cells regulate the RAC1 pathway via ARL4C to promote EMT and mediate oxaliplatin resistance. (A-B)** Mass spectrometry analysis of proteins interacting with ARL4C following ARL4C pull-down. **(C)** 3D molecular docking of ARL4C and RAC1. **(D)** 3D molecular docking of ARL4C and RAP1A. **(E-F)** Dual immunofluorescence staining to detect co-localization of ARL4C with RAC1 and RAP1A proteins. **(G)** CoIP assays to examine the interaction between ARL4C and RAC1/RAP1A proteins. **(H)** Western blot analysis of GTP-RAC1 expression following ARL4C overexpression and MG132 treatment. **(I-M)** Western blot analysis of GTP-RAC1 and Arp2/3 protein expression following ARL4C knockdown/overexpression and oxaliplatin treatment. **(N)** Western blot analysis of Arp2/3 and EMT-related proteins following ARL4C overexpression, oxaliplatin treatment, or RAC1 knockdown. **(O)** Transwell assays to assess the effect of RAC1 knockdown and oxaliplatin treatment on colorectal cancer cell invasion. **(P)** Wound healing assays to evaluate the impact of RAC1 knockdown and oxaliplatin treatment on colorectal cancer cell migration. **(Q-R)** Clonogenic assays to examine the effects of ARL4C overexpression and RAC1 knockdown or AKTi-1/2 treatment on colorectal cancer cell proliferation. **(S-V)** Effects of ARL4C overexpression and RAC1 knockdown on lung metastasis and subcutaneous tumor growth in HCT-116 cells. (* p < 0.05, ** p < 0.01, *** p < 0.001, ns. p > 0.05).

We examined the mechanisms by which ARL4C mediates oxaliplatin resistance in CRC by performing ARL4C pull-down assays followed by mass spectrometry analysis. The results revealed that ARL4C interacts with RAP1A, a key member of the RAP1 family involved in cell adhesion, polarity, and signal transduction (Figure 6A-B). Molecular docking analysis identified multiple binding sites between ARL4C and RAP1A (Figure 6D, Figure S8B, Table S5), and immunofluorescence staining demonstrated their co-localization in CRC cells (Figure 6E). Co-immunoprecipitation (CoIP) assays further confirmed their interaction (Figure 6G). Previous studies have demonstrated that RAP1A activates PI3K, leading to AKT phosphorylation and subsequent mTOR activation, which in turn regulates tumor resistance, proliferation, and survival. Based on these findings, we hypothesized that ARL4C drives oxaliplatin resistance primarily by interacting with RAP1A to activate the ARL4C/RAP1/PI3K-Akt/mTOR signaling axis and modulate its own ubiquitination. RAC1, a central component of the Rho family small GTPase signaling network, plays pivotal roles in cytoskeletal remodeling, migration, invasion, and EMT during tumor progression. Interestingly, ARL4C was also found to interact with RAC1 (Figure 6A-B). This interaction was validated via molecular docking, co-localization studies, and CoIP assays (Figure 6C, F-G; Figure S8A; Table S5). Since both ARL4C and RAC1 belong to the small GTPase family, and ARL4C has been proposed to regulate RAC1 activation via GEFs such as Tiam1 and Vav2, we sought to clarify RAC1's role in ARL4C-mediated oxaliplatin resistance.

We first assessed RAC1-GTP levels in ARL4C-overexpressing cells treated with the proteasome inhibitor MG132. Notably, compared with controls, RAC1-GTP levels were markedly elevated following ARL4C overexpression, MG132 treatment, or their combination (Figure 6H), suggesting that RAC1 signaling is robustly activated, possibly downstream of ARL4C signaling. To further investigate RAC1's role, we knocked down ARL4C and examined RAC1-GTP and its downstream effector, the Arp2/3 complex. Arp2/3, critical for actin branching and cellular motility, is known to drive tumor cell migration and invasion. ARL4C knockdown resulted in a significant reduction in RAC1-GTP and Arp2/3 expression, whereas oxaliplatin treatment increased both markers. Strikingly, co-treatment with ARL4C knockdown and oxaliplatin completely abolished oxaliplatin-induced RAC1 activation (Figure 6I-J, L; Figure S8C), implicating RAC1 as a critical compensatory pathway for oxaliplatin resistance, dependent on ARL4C.

We used ARL4C-overexpressing cells and further confirmed that oxaliplatin treatment in combination with ARL4C overexpression triggered robust RAC1 signaling activation, far exceeding the levels observed with either treatment alone (Figure 6K, M), underscoring ARL4C's essential role in driving RAC1-mediated compensatory resistance mechanisms. Mechanistically, RAC1 activation has been reported to promote EMT through various pathways, including the induction of E-cadherin internalization and degradation, upregulation of N-cadherin, remodeling of F-actin via PAK1/Cortactin/Arp2/3 signaling, and enhancing Vimentin expression 32. These changes collectively endow cells with a mesenchymal phenotype conducive to invasion and metastasis. We observed additional branches in the RAP1/PI3K-Akt/mTOR axis contributing to CRC invasion (Figure 4G-H). To determine whether RAC1 participates in ARL4C-mediated EMT under oxaliplatin pressure, we silenced RAC1 using siRNA. RAC1 knockdown suppressed Arp2/3 expression and significantly impaired EMT (Figure 6N, Figure S8D-G). Notably, silencing RAC1 neutralized the EMT-promoting effects of ARL4C overexpression, oxaliplatin treatment, or their combination, suggesting that RAC1 signaling is essential for ARL4C-driven EMT during oxaliplatin resistance.

We next performed Transwell and wound healing assays, which showed that either RAC1 knockdown or oxaliplatin treatment significantly reduced the migration and invasion of CRC cells. However, combined treatment produced a synergistic inhibition of metastasis (Figure 6O-P; Figure S8H, K), suggesting that RAC1 depletion sensitizes CRC cells to oxaliplatin. Although RAC1 primarily governs migration, it also plays a modest role in proliferation. Colony formation assays revealed that RAC1 knockdown had a minimal effect on proliferation compared to AKTi1/2-mediated inhibition (Figure 6Q-R, Figure S8I-J), suggesting that RAC1 signaling primarily regulates the metastatic rather than the proliferative phenotype. Finally, *in vivo* experiments demonstrated that RAC1 knockdown markedly reduced ARL4C-driven pulmonary metastasis without significantly impacting subcutaneous tumor growth (Figure 6S-V). These findings were consistent with our *in vitro* results. Collectively, these data demonstrated that CRC cells exploit ARL4C-mediated activation of the RAC1 signaling pathway to promote EMT and confer resistance to oxaliplatin.

### β-Lapachone Targets ARL4C to Synergistically Suppress CRC Progression in Combination with Oxaliplatin

**Figure 7:**
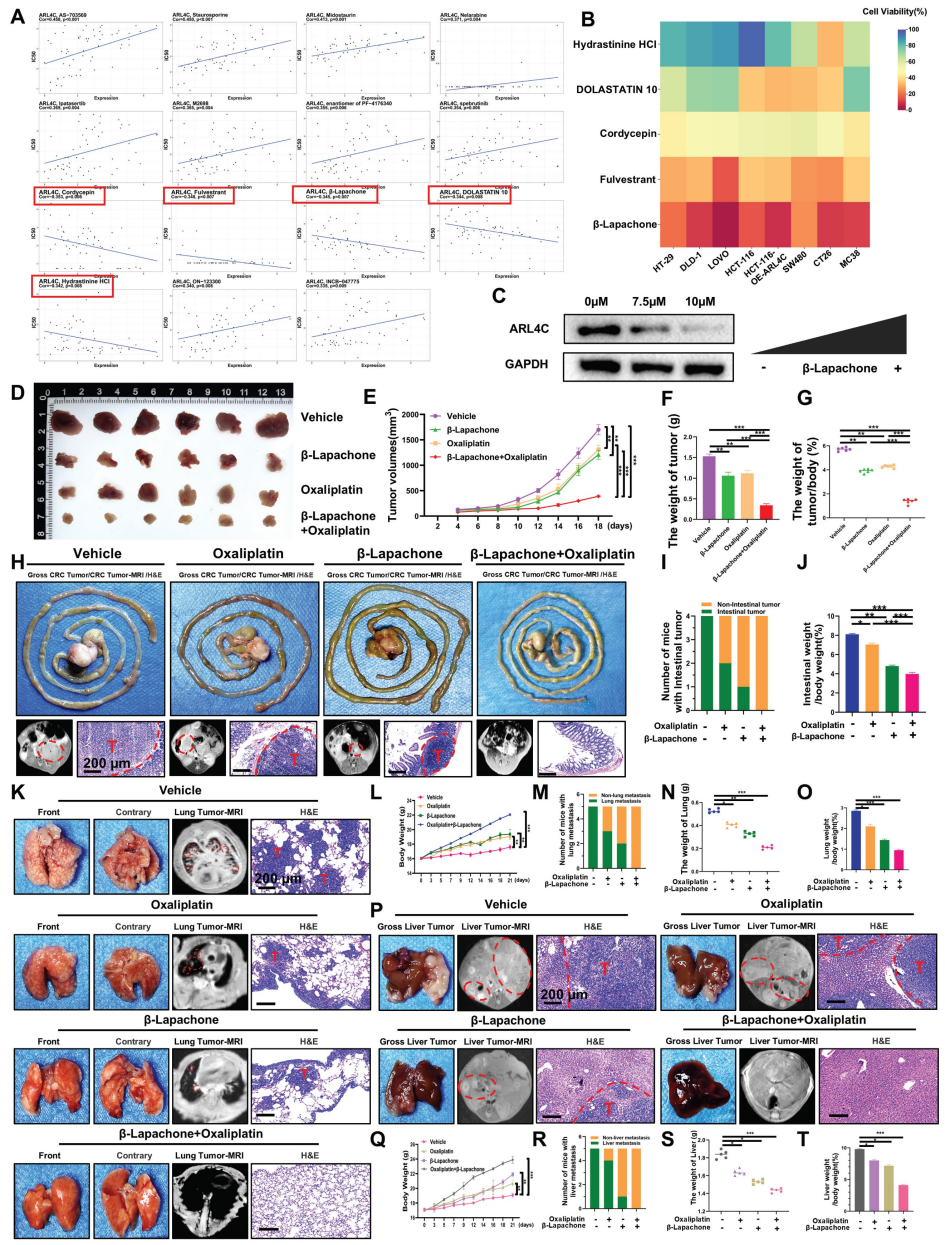
** β-Lapachone targets ARL4C to synergistically inhibit colorectal cancer progression in combination with oxaliplatin. (A)** IC50 values of 15 small molecules screened through DrugBank, ChEMBL, and other databases, and their correlation with ARL4C expression levels. **(B)** Effect of the 5 selected ARL4C small molecule inhibitors on cell viability in six human colorectal cancer cell lines (HT-29, DLD-1, LOVO, HCT-116, HCT-116-OE-ARL4C, SW480) and two murine colorectal cancer cell lines (CT-26, MC38). **(C)** Western blot analysis of ARL4C protein expression in colorectal cancer cell lines treated with different concentrations of β-Lapachone. **(D-G)** Effects of β-Lapachone and oxaliplatin treatment on CT-26 subcutaneous tumor growth. **(H)** Effects of β-Lapachone and oxaliplatin treatment on CT-26 orthotopic colon tumor growth. **(I)** Number of Balb/c mice with intestinal tumors. **(J)** Ratio of intestinal weight to body weight in the colon orthotopic syngeneic xenograft model. **(K)** Effects of β-Lapachone and oxaliplatin treatment on CT-26 lung metastasis. **(L)** Changes in body weight of mice from different treatment groups over time. **(M)** Number of mice with or without lung metastasis in each group. **(N)** Lung weight in each treatment group. **(O)** Lung weight to body weight ratio in each treatment group. **(P-T)** Effects of β-Lapachone and oxaliplatin treatment on CT-26 liver metastasis. (* p < 0.05, ** p < 0.01, *** p < 0.001, ns. p > 0.05).

ARL4C plays a pivotal role in oxaliplatin resistance in CRC, and targeting ARL4C may represent a promising strategy to overcome chemoresistance. To identify potential ARL4C inhibitors, we employed a network pharmacology approach integrated with computational modeling, bioinformatics, and systems biology. By mining DrugBank, ChEMBL, and other databases, we identified 15 small-molecule compounds strongly associated with ARL4C. Among them, five candidates—including Cordycepin, Fulvestrant, β-Lapachone, DOLASTATIN 10, and Hydrastinine HCl—showed negative correlations with ARL4C expression (Figure 7A). To evaluate their inhibitory potential, these five compounds were tested on HCT116 cells overexpressing ARL4C, as well as on commonly used human and murine CRC cell lines. β-Lapachone exhibited the most potent and consistent antitumor activity across all models (Figure 7B). Notably, ARL4C overexpression significantly increased the sensitivity of HCT116 cells to β-Lapachone. Building on our prior *in vivo* data—where ARL4C was shown to modulate oxaliplatin sensitivity in human CRC cell lines using a nude mouse model—we sought to validate these findings in a syngeneic system. To eliminate interspecies transplantation-related artifacts and enhance physiological relevance, we used Balb/c mice and the murine colon cancer cell line CT-26. Treatment of CT-26 cells with increasing concentrations of β-Lapachone resulted in a dose-dependent reduction of ARL4C expression (Figure 7C), further confirming β-Lapachone as a functional ARL4C inhibitor capable of exerting anticancer effects via ARL4C suppression.

We established subcutaneous tumor models in Balb/c mice to explore the therapeutic potential of β-Lapachone against oxaliplatin-resistant CRC, and administered β-Lapachone, oxaliplatin, or their combination (Figure 7D). Both monotherapies moderately inhibited tumor growth, whereas the combination treatment markedly suppressed tumor progression (Figure 7E-G), demonstrating a strong synergistic antitumor effect. We further validated these findings using an orthotopic colon tumor model (Figure 7H). While either β-Lapachone or oxaliplatin alone modestly inhibited tumor growth, combination therapy completely prevented tumor implantation and expansion (Figure 7I-J), highlighting a more pronounced therapeutic advantage in orthotopic settings compared to subcutaneous models.

Building on our earlier findings that ARL4C knockdown significantly suppresses CRC metastasis in combination with oxaliplatin, we further investigated whether β-Lapachone could substitute for ARL4C depletion to achieve similar anti-metastatic effects. Cancer cachexia, a systemic metabolic syndrome characterized by weight loss and muscle/fat wasting despite adequate nutrition, was also assessed 33. Using 4-week-old Balb/c mice, we observed that tumor-bearing mice exhibited impaired weight gain compared to the treated groups (Figure 7K-L). Treatment with β-Lapachone and/or oxaliplatin significantly reduced lung metastatic nodules (Figure 7M-O). Remarkably, combination therapy completely abrogated lung metastasis, demonstrating that β-Lapachone could effectively substitute for ARL4C knockdown when combined with oxaliplatin, offering promising translational value. To further validate the stability of β-Lapachone as an ARL4C inhibitor, we established a liver metastasis model (Figure 7P). Consistently, β-Lapachone monotherapy significantly inhibited liver metastasis compared to controls and oxaliplatin alone, while combination treatment entirely prevented metastatic colonization (Figure 7Q-T).

**Figure 8:**
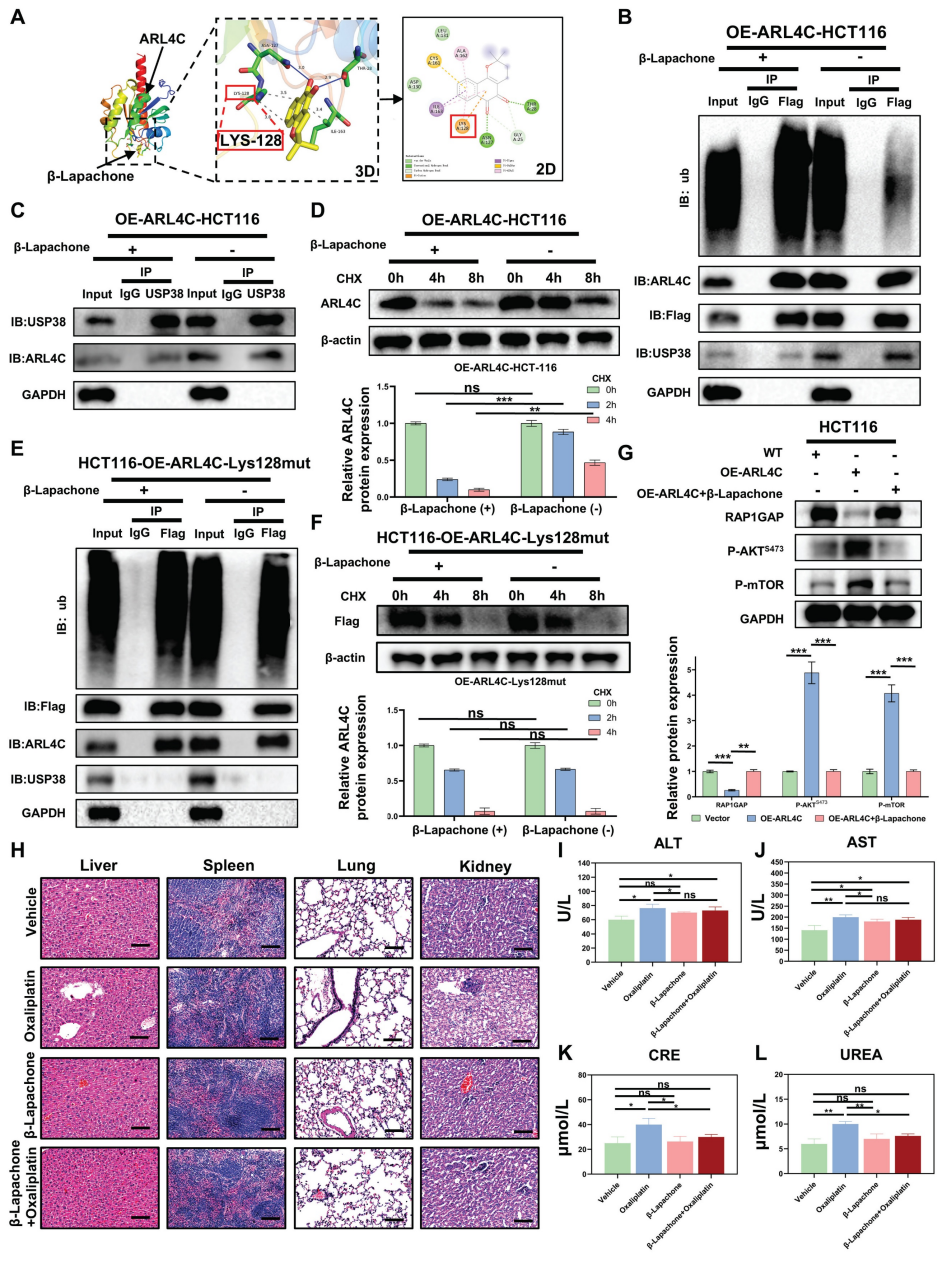
** β-Lapachone competitively binds to ARL4C with USP38, promoting the ubiquitination and degradation of ARL4C. (A)** 2D and 3D molecular docking of β-Lapachone with ARL4C. **(B)** Co-IP analysis of ARL4C ubiquitination levels under β-Lapachone treatment. **(C)** Co-IP analysis of the interaction between ARL4C and USP38 under β-Lapachone treatment. **(D)** Western Blot analysis of the effect of β-Lapachone on ARL4C degradation rate under CHX treatment. **(E)** Co-IP analysis of the effect of β-Lapachone on ARL4C and USP38 interaction in the case of Lys128 mutation in ARL4C. **(F)** Western Blot analysis of the effect of β-Lapachone on ARL4C degradation rate under CHX treatment in the case of Lys128 mutation in ARL4C. **(G)** Western Blot analysis of the effect of β-Lapachone on the RAP1/PI3K-Akt-mTOR signaling loop. **(H)** H&E staining of major organs (liver, spleen, lung, kidney) under different treatments. **(I-L)** Serum biochemical analysis after vehicle, oxaliplatin, β-Lapachone, or combination therapy. (* p < 0.05, ** p < 0.01, *** p < 0.001, ns. p > 0.05).

Molecular docking experiments revealed that β-Lapachone has several potential binding sites on ARL4C. Interestingly, we found that β-Lapachone binds strongly to the Lys128 site of ARL4C (Figure 8A). Upon treatment with β-Lapachone, the protein expression levels of both ARL4C and USP38 were downregulated, and their interaction was significantly weakened (Figure 8B-C). Additionally, treatment with β-Lapachone led to a marked increase in ARL4C's ubiquitination levels, and its degradation rate was accelerated considerably (Figure 8B, D). We further explored the mechanism by which β-Lapachone promotes ARL4C ubiquitination and degradation by investigating its effect in the ARL4C Lys128 mutant. We found that, with the Lys128 site mutation, β-Lapachone lost its regulatory impact on exogenous ARL4C, but still exerted control over endogenous ARL4C (Figure 8E-F). This suggested that β-Lapachone exerts its effect by competitively binding to the Lys128 site with USP38, thereby promoting ARL4C's ubiquitination and degradation. Consistent with this conclusion, treatment with β-Lapachone reversed the regulatory effect of ARL4C overexpression on the RAP1/PI3K-Akt/mTOR signaling pathway (Figure 8G). It has been previously reported that NQO1-dependent DNA damage is a common mechanism by which β-Lapachone exerts its effects 34. To investigate whether NQO1 is involved in the synergistic effect of β-Lapachone and oxaliplatin, we constructed NQO1 knockdown cell lines and found that the knockdown of NQO1 did not affect the synergistic effect of β-Lapachone and oxaliplatin (Figure S9A).

We comprehensively evaluated the antitumor efficacy and systemic toxicity of different therapeutic strategies by conducting histological and biochemical analyses in CRC xenograft-bearing mice. Histopathological examination of major organs (liver, spleen, lungs, and kidneys) using hematoxylin and eosin (H&E) staining revealed no overt structural abnormalities across treatment groups, likely due to the relatively short duration of exposure. Notably, in the oxaliplatin-treated group, no evidence of cellular necrosis or inflammatory infiltration was observed, suggesting the absence of gross organ damage at the tissue level. However, serum biochemical analysis indicated elevated levels of ALT, AST, creatinine (CRE), and urea (UREA) following oxaliplatin administration, reflecting early-stage hepatic and renal functional impairment (Figure 8H-L). Strikingly, co-administration of β-Lapachone with oxaliplatin markedly attenuated these elevations in CRE and UREA, restoring their levels closer to those of control mice, suggesting that β-Lapachone does not exacerbate oxaliplatin-induced toxicity and may also confer renal protection. Significantly, this combination also elicited a pronounced synergistic effect in suppressing tumor growth, underscoring its therapeutic potential. Taken together, these findings identified β-Lapachone as a potent ARL4C inhibitor that, when combined with oxaliplatin, exerts a robust synergistic antitumor effect while mitigating oxaliplatin-associated nephrotoxicity, thus offering a favorable safety profile and promising translational potential.

In summary, through network pharmacology screening, β-Lapachone emerged as a potent ARL4C inhibitor, promoting ARL4C degradation by competitively binding to the LYS128 site targeted by deubiquitinases. Functionally, β-Lapachone in combination with oxaliplatin robustly suppressed tumor metastasis, comparable to ARL4C knockdown plus oxaliplatin. This strategy amplified oxaliplatin's anti-metastatic efficacy and offered a novel approach to overcoming chemoresistance, providing solid preclinical evidence supporting ARL4C as a therapeutic target for metastatic CRC.

### Elevated ARL4C Expression Correlates with Postoperative Recurrence and Distant Metastasis in Oxaliplatin-Treated Colorectal Cancer Patients

**Figure 9:**
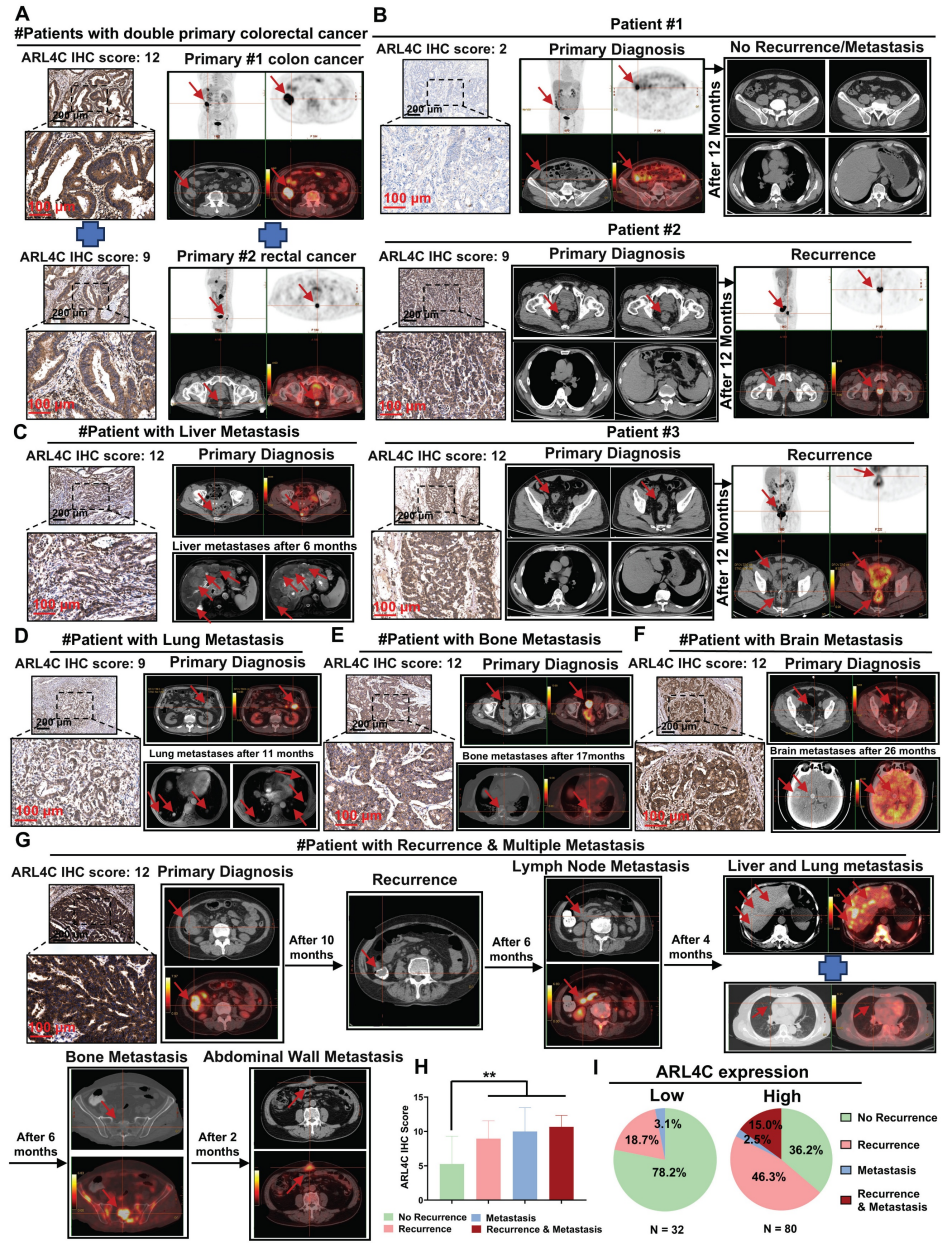
** ARL4C involvement in colorectal cancer onset, progression, recurrence, and distant metastasis. (A)** Immunohistochemical detection of ARL4C expression and imaging evidence in dual primary colorectal cancer. **(B)** Immunohistochemical detection of ARL4C expression and imaging evidence in recurrent and non-recurrent colorectal cancer. **(C)** Immunohistochemical detection of ARL4C expression and imaging evidence in colorectal cancer with liver metastasis. **(D)** Immunohistochemical detection of ARL4C expression and imaging evidence in colorectal cancer with lung metastasis. **(E)** Immunohistochemical detection of ARL4C expression and imaging evidence in colorectal cancer with bone metastasis. **(F)** Immunohistochemical detection of ARL4C expression and imaging evidence in colorectal cancer with brain metastasis. **(G)** Immunohistochemical detection of ARL4C expression and imaging data in the same patient showing sequential recurrence, lymph node metastasis, liver-lung metastasis, bone metastasis, and abdominal wall metastasis. **(H-I)** Correlation of ARL4C expression with colorectal cancer metastasis or recurrence. (* p < 0.05, ** p < 0.01, *** p < 0.001, ns. p > 0.05).

Based on our earlier mechanistic investigations, we further analyzed clinical specimens and imaging data from 112 patients with CRC who received oxaliplatin-based chemotherapy. We aimed to elucidate the expression patterns of ARL4C during CRC progression and metastasis and to assess its association with patient prognosis. Clinically, patients with synchronous double primary tumors generally exhibit worse outcomes compared to those with solitary tumors, and their prognosis is typically poorer than that of those with metachronous double primaries. Interestingly, we found that ARL4C expression was markedly elevated in synchronous double primary CRC tumors (Figure 9A). Similarly, ARL4C was highly expressed in primary and metastatic lesions from patients with synchronous colorectal liver metastasis (Figure S10A). Immunohistochemical analysis of 112 clinical specimens revealed that ARL4C expression was significantly higher in tumors from patients who experienced recurrence compared to those without recurrence following oxaliplatin treatment (Figure 9B). These findings suggested that ARL4C upregulation may contribute to oxaliplatin resistance and subsequent tumor relapse. Moreover, in four representative cases exhibiting distant metastases—liver metastasis at 6 months, pulmonary metastasis at 11 months, bone metastasis at 17 months, and brain metastasis at 26 months post-surgery—ARL4C levels were markedly elevated (Figure 9C-F). These observations implicated ARL4C as a critical driver of metastatic dissemination in CRC.

Of particular interest, we identified one patient who developed sequential intraluminal recurrence, lymph node metastasis, liver-lung metastases, bone metastasis, and abdominal wall metastasis over a 28-month follow-up period despite receiving oxaliplatin-based chemotherapy. Tumor samples from this patient exhibited substantially higher ARL4C expression compared to other cases with either isolated recurrence or single-site metastasis (Figure 9G). Quantitative analysis revealed that CRC patients with high ARL4C expression were significantly more likely to experience recurrence (61.3% vs. 18.7%) and metastasis (17.5% vs. 3.1%) than those with low ARL4C expression following oxaliplatin-based therapy (Figure 9H-I).

To further stratify prognosis, we classified the 112 patients into four groups based on the relative expression levels of ARL4C and RAC1: high ARL4C/high RAC1, high ARL4C/low RAC1, low ARL4C/high RAC1, and low ARL4C/low RAC1 (Figure S10B). Survival analysis demonstrated that patients with low ARL4C and low RAC1 expression exhibited significantly better outcomes than those with high ARL4C and high RAC1 expression (Figure S10C-D), corroborating our mechanistic hypotheses. This conclusion was further validated using large-scale TCGA dataset analyses (Figure S10E). Together, these results underscored ARL4C as a critical biomarker for predicting recurrence and distant metastasis in CRC patients undergoing oxaliplatin-based chemotherapy, highlighting its potential as a therapeutic target to overcome chemoresistance and metastasis.

## Discussion

This study systematically elucidated ARL4C, a member of the small GTPase family, as a dual driver of oxaliplatin resistance and metastasis in CRC. Our data suggested that ARL4C has strong biomarker potential for patient stratification in CRC. IHC analysis revealed that ARL4C expression was significantly elevated in resistant patients and strongly correlated with tumor grade, stage, recurrence, and metastasis. Notably, higher ARL4C levels were observed in aggressive subtypes, such as mucinous adenocarcinoma. These findings support the use of ARL4C expression via IHC or liquid biopsy to identify high-risk patients and guide clinical decision-making. Multi-cohort survival analyses indicated that high ARL4C expression was also associated with worse OS, PFS, and RFS, supporting its role as a potential independent prognostic marker. Functional assays demonstrated that ARL4C knockdown sensitized CRC cells to oxaliplatin and promoted apoptosis, whereas ARL4C overexpression accelerated tumor growth and impaired chemotherapy response *in vivo*. In metastatic models, ARL4C overexpression markedly increased distant metastases, predominantly in the liver and lungs. Mechanistically, ARL4C acted as a "central integrator," actively promoting both drug resistance and metastatic progression, establishing a cooperative model of "resistance plus metastasis." Our findings provide new insights into therapeutic resistance and recurrence in advanced CRC and highlight ARL4C as a promising target for clinical intervention.

We further elucidated that the ARL4C/RAP1/PI3K-Akt-mTOR signaling loop governs CRC cell survival and oxaliplatin resistance. ARL4C overexpression robustly activated RAP1, sustained PI3K, p-Akt, and p-mTOR levels, enhanced chemoresistance, and suppressed apoptosis, accompanied by Bcl-2 upregulation and Bax downregulation. Notably, ARL4C activated this pathway to maintain its own protein stability, forming a positive feedback loop of "signal amplification-protein stabilization-sustained resistance." Pharmacological inhibition of Akt or mTOR markedly reduced ARL4C expression and reversed the resistant phenotype, validating this dynamic maintenance mechanism. Thus, ARL4C establishes a self-sustaining resistance network in CRC through dual mechanisms of functional activation and expression maintenance.

Additionally, we demonstrated that ARL4C stabilizes its protein levels by regulating the deubiquitination system, reinforcing its role in driving resistance and metastasis. Protein homeostasis is not only controlled by ubiquitination via E3 ligases but also critically dependent on DUBs that remove ubiquitin chains. ARL4C overexpression was accompanied by significant upregulation of multiple DUB family members, suggesting that ARL4C evades proteasomal degradation via enhanced deubiquitination. Particularly under chemotherapy-induced stress, ARL4C utilized DUB-mediated "immune evasion" to maintain protein integrity. This mechanism reveals that ARL4C is not merely a signaling activator but also employs self-protective strategies to sustain expression within the tumor microenvironment, offering a novel rationale for therapeutic targeting and highlighting DUBs as alternative intervention points.

Our findings further revealed that ARL4C promoted EMT through RAC1 activation, markedly enhancing CRC cell migration and invasion. Subcutaneous tumor and metastasis models confirmed that ARL4C overexpression significantly increased metastatic nodules in the lungs and liver, while ARL4C silencing nearly abolished metastasis formation. Mechanistically, ARL4C activated RAC1, downregulated E-cadherin, and upregulated Vimentin, leading to a classical EMT molecular signature and enhanced cellular motility and distant colonization. RAC1, a key regulator of cytoskeletal remodeling and polarity conversion, also conferred greater resistance to oxaliplatin-induced stress 35-37. Notably, EMT status was associated with heightened chemoresistance 38-40. Our results were consistent with those of Huang et al. 41-43, and indicated that EMT contributes to oxaliplatin resistance. However, we acknowledge that EMT may exert dual effects on the chemotherapeutic response, as other studies have suggested that EMT can suppress cell proliferation and enhance sensitivity to DNA-damaging agents. Therefore, EMT should not be viewed as a uniform driver of resistance. Instead, its role may vary depending on cellular context and tumor type. This dual role of EMT has been shown to better reflect its complex involvement in chemoresistance. Our research found that ARL4C orchestrates migration and drug resistance via the RAC1-EMT axis, uncovering a pivotal mechanism underlying treatment-refractory CRC and providing a theoretical basis for combined anti-metastatic and anti-resistance strategies.

In this study, β-Lapachone combined with oxaliplatin exhibited a potent synergistic antitumor effect. Given the critical role of ARL4C in CRC resistance and metastasis, targeting ARL4C emerged as a key therapeutic strategy. Through network pharmacology and molecular docking analyses across multiple drug-target databases, we identified β-Lapachone, a natural naphthoquinone derived from Tabebuia species 44-46, as the most promising ARL4C inhibitor candidate. Mechanistic studies suggested that β-Lapachone may competitively bind to the LYS128 site of ARL4C, enhancing its degradation and suppressing downstream signaling. *In vitro*, β-Lapachone significantly lowered oxaliplatin IC50, potentiated apoptosis, and synergistically inhibited migration and invasion. The combination therapy substantially reduced tumor volumes and metastases *in vivo* while mitigating oxaliplatin-induced nephrotoxicity. Histological analyses confirmed extensive nuclear pyknosis and necrosis in tumors from the combination group. Remarkably, treated mice maintained normal liver, spleen, lung, and kidney function, indicating excellent safety. Collectively, the β-Lapachone/oxaliplatin combination leverages a "target inhibition plus DNA damage" strategy, exhibiting potent synergy and strong translational potential, particularly in ARL4C-high, oxaliplatin-resistant CRC.

Currently, CRC treatment primarily relies on surgical resection and oxaliplatin-based chemotherapy, with targeted therapies limited to anti-VEGF (bevacizumab) and anti-EGFR (cetuximab) antibodies 47-49. Although immunotherapy has demonstrated efficacy in patients with MSI-H/dMMR CRC, its clinical utility remains limited due to the low prevalence of this molecular subtype, which restricts its broader applicability 50-52. While existing therapeutic strategies offer benefits in select patient populations, they remain largely ineffective in controlling disease progression and metastasis in individuals with intrinsic drug resistance or high metastatic potential. This limitation is particularly evident in patients with elevated ARL4C expression, for whom conventional treatments often fail to suppress tumor growth and dissemination. Our study proposes an innovative "ARL4C inhibition plus oxaliplatin" strategy that simultaneously disrupts resistance signaling and induces DNA damage, bypassing reliance on RAS/BRAF mutation or MSI/MMR status and expanding clinical applicability. ARL4C regulates both tumor cell survival and metastatic capacity, making it an ideal therapeutic target. Its stability, maintained through deubiquitination, further enhances its druggability. β-Lapachone's favorable oral bioavailability and low toxicity profile increase the accessibility and clinical potential of this combination approach. Overall, this dual-targeting strategy demonstrates compelling advantages in mechanism, target universality, safety, and translational potential.

Despite systematically delineating ARL4C's pivotal role in oxaliplatin resistance and metastasis and proposing a promising translational strategy, our study has limitations. First, the broad target spectrum of β-Lapachone warrants further characterization to optimize specificity and dosing. Second, although classical animal models were employed, future studies should validate the combination strategy using organoid or patient-derived xenograft models to better assess microenvironmental influences. Finally, although β-Lapachone exhibits promising antitumor activity, its clinical translation has been hindered by several pharmacological limitations, including a short plasma half-life and dose-limiting toxicities such as methemoglobinemia. These challenges remain major obstacles to its therapeutic development. To overcome them, a range of tumor-targeted delivery strategies—such as nanoparticle formulations and prodrug designs—are currently being explored. These approaches have demonstrated encouraging results in preclinical studies by improving bioavailability and minimizing off-target toxicity. Moreover, emerging evidence suggests that β-Lapachone exhibits a relatively favorable safety profile, extending beyond its anticancer effects, including anti-inflammatory, antioxidant, renoprotective, and antimicrobial properties, which further supports its potential for rational therapeutic optimization 53. Overall, our study provides a new theoretical framework for precision CRC therapy, with strong prospects for clinical translation.

**Figure 10:**
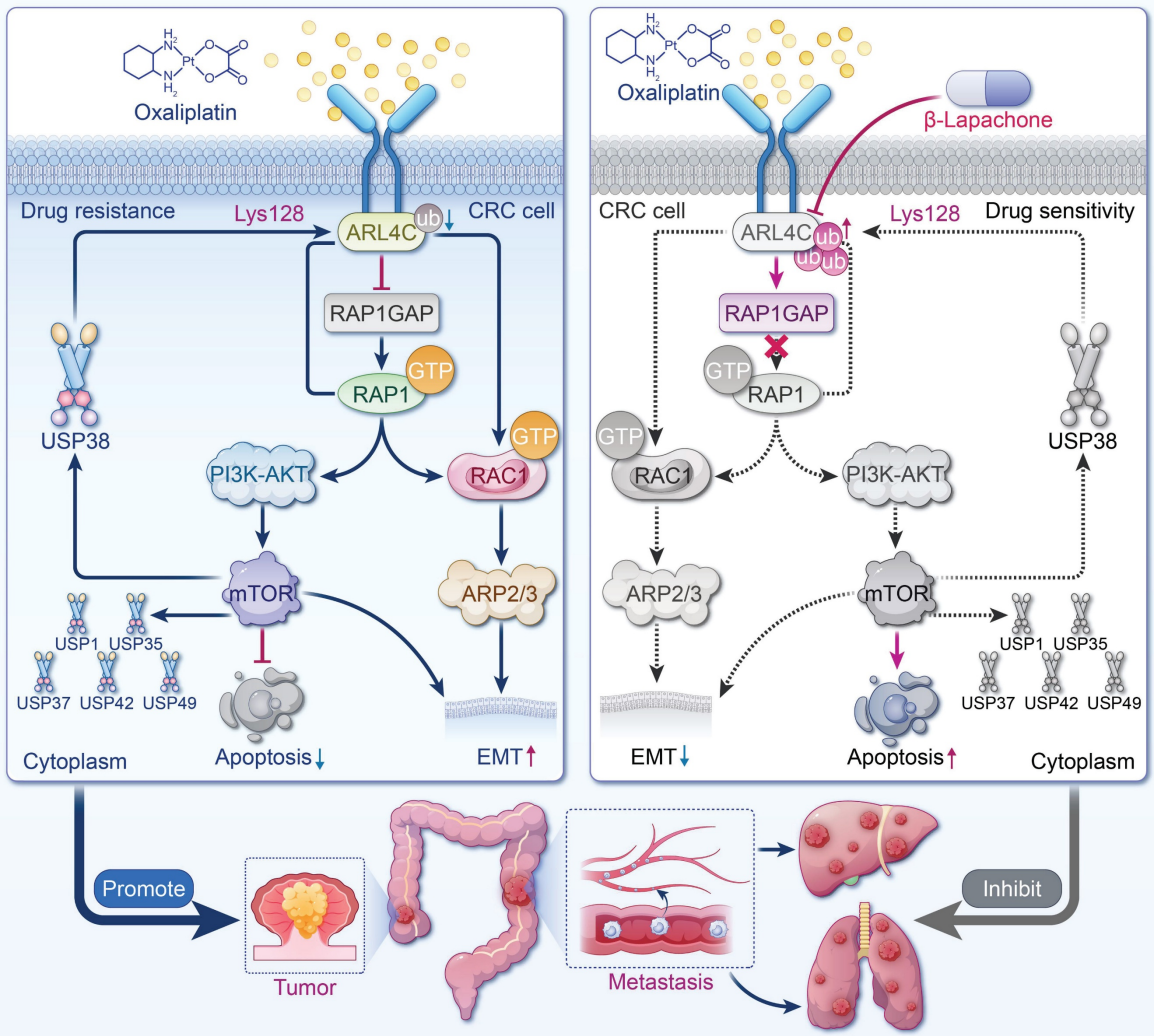
** Graphic summary of ARL4C/RAP1/PI3K-Akt-mTOR signaling loop.** ARL4C drives oxaliplatin resistance and metastasis in CRC via RAP1/PI3K-Akt/mTOR and RAC1/Arp2/3 signaling, stabilized by deubiquitination, establishing a stable positive feedback loop. β-Lapachone disrupts this loop by targeting ARL4C, enhancing chemosensitivity and reducing metastasis.

In conclusion, we have identified ARL4C as a key regulator of oxaliplatin resistance and metastasis in CRC. ARL4C promotes tumor survival and migration through the RAP1/PI3K-Akt/mTOR signaling loop and the RAC1/EMT signaling axis, and sustains its expression via deubiquitinase-mediated stabilization, thereby forming a positive feedback loop. We further discovered that β-Lapachone is a potential inhibitor of ARL4C, promoting its degradation by competitively binding to the LYS128 site of ARL4C with USP38 (Figure 10). Combination therapy with β-Lapachone and oxaliplatin has demonstrated synergistic antitumor effects and reduced toxicity, offering a promising strategy for precision therapy and resistance management in CRC.

## Materials and Methods

### Cell Culture and Genetic Manipulation

Human colorectal cancer cell lines (DLD-1, LOVO, HT-29, SW620, SW480, HCT-116), normal colon epithelial cells (NCM460), murine colorectal cancer cell lines (CT26, MC38), and HEK293T cells were obtained from certified repositories and cultured in RPMI-1640 or DMEM supplemented with 10% fetal bovine serum and 1% penicillin-streptomycin. ARL4C knockdown and overexpression models were established via lentiviral transduction. For knockdown, DLD-1 and LOVO cells were infected with lentiviruses carrying shRNA against ARL4C; HCT-116 cells were transduced with overexpression vectors. Infected cells were selected with 2 µg/mL puromycin. Transfection efficiency was confirmed by qRT-PCR and Western blotting prior to functional assays.

### *In Vivo* Animal Models

All animal procedures were approved by the Laboratory Animal Ethics Committee of the Fourth Affiliated Hospital of Harbin Medical University. BALB/c and BALB/c nude mice (6-8 weeks old) were housed under specific pathogen-free conditions. For subcutaneous models, HCT-116 (ARL4C overexpression or control) or CT26 cells were injected into the flank. Mice received oxaliplatin and/or β-Lapachone intraperitoneally, and tumor growth was monitored every three days. Tumors were harvested after three weeks for histological and molecular analyses. For orthotopic implantation, tumor cells were injected into the cecal wall to mimic native colorectal tumor growth. Lung metastasis was induced via tail vein injection, and liver metastasis via splenic injection. Tumor burden and metastatic lesions were evaluated using MRI, histopathology, and quantification of nodules. Serum biochemistry and H&E staining were used to assess systemic toxicity and organ damage.

## Supplementary Material

Supplementary materials and methods, figures and tables.

## Abbreviations

CRC: colorectal cancer
ARF: ADP-ribosylation factor
ARL4C: ADP-ribosylation factor-like 4C
EMT: epithelial-mesenchymal transition
DUBs: deubiquitinating enzymes
PI3K: phosphoinositide 3-kinase
mTOR: mechanistic target of rapamycin kinase
GO: Gene Ontology
KEGG: Kyoto Encyclopedia of Genes and Genomes
CoIP: Co-immunoprecipitation assay
EdU: 5-Ethynyl-2′-deoxyuridine
OS: overall survival
RFS: Recurrence free survival
KM: Kaplan-Meier

## References

[1] Bray F, Laversanne M, Sung H, Ferlay J, Siegel RL, Soerjomataram I (2024). Global cancer statistics 2022: GLOBOCAN estimates of incidence and mortality worldwide for 36 cancers in 185 countries. CA Cancer J Clin.

[2] Shaukat A, Levin TR (2022). Current and future colorectal cancer screening strategies. Nat Rev Gastroenterol Hepatol.

[3] Wang L, Tu YX, Chen L, Zhang Y, Pan XL, Yang SQ (2023). Male-Biased Gut Microbiome and Metabolites Aggravate Colorectal Cancer Development. Adv Sci (Weinh).

[4] Heinemann V, Stintzing S (2024). Liver transplantation in metastatic colorectal cancer: a new standard of care?. Lancet.

[5] Wasan HS, Gibbs P, Sharma NK, Taieb J, Heinemann V, Ricke J (2017). First-line selective internal radiotherapy plus chemotherapy versus chemotherapy alone in patients with liver metastases from colorectal cancer (FOXFIRE, SIRFLOX, and FOXFIRE-Global): a combined analysis of three multicentre, randomised, phase 3 trials. Lancet Oncol.

[6] Schmoll HJ, Stein A, Van Cutsem E, Price T, Hofheinz RD, Nordlinger B (2021). Pre- and Postoperative Capecitabine Without or With Oxaliplatin in Locally Advanced Rectal Cancer: PETACC 6 Trial by EORTC GITCG and ROG, AIO, AGITG, BGDO, and FFCD. J Clin Oncol.

[7] Tournigand C, Andre T, Achille E, Lledo G, Flesh M, Mery-Mignard D (2023). FOLFIRI Followed by FOLFOX6 or the Reverse Sequence in Advanced Colorectal Cancer: A Randomized GERCOR Study. J Clin Oncol.

[8] Hashemi M, Esbati N, Rashidi M, Gholami S, Raesi R, Bidoki SS (2024). Biological landscape and nanostructural view in development and reversal of oxaliplatin resistance in colorectal cancer. Transl Oncol.

[9] Rottenberg S, Disler C, Perego P (2021). The rediscovery of platinum-based cancer therapy. Nat Rev Cancer.

[10] Linares J, Sallent-Aragay A, Badia-Ramentol J, Recort-Bascuas A, Mendez A, Manero-Ruperez N (2023). Long-term platinum-based drug accumulation in cancer-associated fibroblasts promotes colorectal cancer progression and resistance to therapy. Nat Commun.

[11] Daly W, Lakhoua G, Zaiem A, Charfi O, Kastalli S, Daghfous R (2023). Atypical neurological toxicity attributed to oxaliplatin. Therapie.

[12] Combes E, Andrade AF, Tosi D, Michaud HA, Coquel F, Garambois V (2019). Inhibition of Ataxia-Telangiectasia Mutated and RAD3-Related (ATR) Overcomes Oxaliplatin Resistance and Promotes Antitumor Immunity in Colorectal Cancer. Cancer Res.

[13] Gu Z, Yin J, Da Silva CG, Liu Q, Cruz LJ, Ossendorp F (2024). Therapeutic liposomal combination to enhance chemotherapy response and immune activation of tumor microenvironment. J Control Release.

[14] Iden S, Collard JG (2008). Crosstalk between small GTPases and polarity proteins in cell polarization. Nat Rev Mol Cell Biol.

[15] Messina S, De Simone G, Ascenzi P (2019). Cysteine-based regulation of redox-sensitive Ras small GTPases. Redox Biol.

[16] Maldonado MDM, Dharmawardhane S (2018). Targeting Rac and Cdc42 GTPases in Cancer. Cancer Res.

[17] Kim WY, Sharpless NE (2006). The regulation of INK4/ARF in cancer and aging. Cell.

[18] Gillingham AK, Munro S (2007). The small G proteins of the Arf family and their regulators. Annu Rev Cell Dev Biol.

[19] Harada A, Matsumoto S, Yasumizu Y, Shojima K, Akama T, Eguchi H (2021). Localization of KRAS downstream target ARL4C to invasive pseudopods accelerates pancreatic cancer cell invasion. Elife.

[20] Zhang J, Zhang Q, Sun C, Huang Y, Zhang J, Wang Q (2020). Clinical relevance of ARF/ARL family genes and oncogenic function of ARL4C in endometrial cancer. Biomed Pharmacother.

[21] Conroy T, Bosset JF, Etienne PL, Rio E, Francois E, Mesgouez-Nebout N (2021). Neoadjuvant chemotherapy with FOLFIRINOX and preoperative chemoradiotherapy for patients with locally advanced rectal cancer (UNICANCER-PRODIGE 23): a multicentre, randomised, open-label, phase 3 trial. Lancet Oncol.

[22] Schrag D, Shi Q, Weiser MR, Gollub MJ, Saltz LB, Musher BL (2023). Preoperative Treatment of Locally Advanced Rectal Cancer. N Engl J Med.

[23] Stordal B, Pavlakis N, Davey R (2007). Oxaliplatin for the treatment of cisplatin-resistant cancer: a systematic review. Cancer Treat Rev.

[24] Mauri G, Gori V, Bonazzina E, Amatu A, Tosi F, Bencardino K (2020). Oxaliplatin retreatment in metastatic colorectal cancer: Systematic review and future research opportunities. Cancer Treat Rev.

[25] Bernal Astrain G, Strakhova R, Jo CH, Teszner E, Killoran RC, Smith MJ (2025). The small GTPase MRAS is a broken switch. Nat Commun.

[26] Crosas-Molist E, Samain R, Kohlhammer L, Orgaz JL, George SL, Maiques O (2022). Rho GTPase signaling in cancer progression and dissemination. Physiol Rev.

[27] Han JS, Hino K, Li W, Reyes RV, Canales CP, Miltner AM (2020). CRL5-dependent regulation of the small GTPases ARL4C and ARF6 controls hippocampal morphogenesis. Proc Natl Acad Sci U S A.

[28] Oliveira RC, Abrantes AM, Tralhao JG, Botelho MF (2020). The role of mouse models in colorectal cancer research-The need and the importance of the orthotopic models. Animal Model Exp Med.

[29] Glaviano A, Foo ASC, Lam HY, Yap KCH, Jacot W, Jones RH (2023). PI3K/AKT/mTOR signaling transduction pathway and targeted therapies in cancer. Mol Cancer.

[30] Shah S, Brock EJ, Ji K, Mattingly RR (2019). Ras and Rap1: A tale of two GTPases. Semin Cancer Biol.

[31] Ma L, Zhang R, Li D, Qiao T, Guo X (2021). Fluoride regulates chondrocyte proliferation and autophagy via PI3K/AKT/mTOR signaling pathway. Chem Biol Interact.

[32] Gulhati P, Bowen KA, Liu J, Stevens PD, Rychahou PG, Chen M (2011). mTORC1 and mTORC2 regulate EMT, motility, and metastasis of colorectal cancer via RhoA and Rac1 signaling pathways. Cancer Res.

[33] Berriel Diaz M, Rohm M, Herzig S (2024). Cancer cachexia: multilevel metabolic dysfunction. Nat Metab.

[34] Bey EA, Bentle MS, Reinicke KE, Dong Y, Yang CR, Girard L (2007). An NQO1- and PARP-1-mediated cell death pathway induced in non-small-cell lung cancer cells by beta-lapachone. Proc Natl Acad Sci U S A.

[35] Toyama Y, Kontani K, Katada T, Shimada I (2019). Decreased conformational stability in the oncogenic N92I mutant of Ras-related C3 botulinum toxin substrate 1. Sci Adv.

[36] Liu L, Cui J, Zhao Y, Liu X, Chen L, Xia Y (2021). KDM6A-ARHGDIB axis blocks metastasis of bladder cancer by inhibiting Rac1. Mol Cancer.

[37] Myant KB, Cammareri P, McGhee EJ, Ridgway RA, Huels DJ, Cordero JB (2013). ROS production and NF-kappaB activation triggered by RAC1 facilitate WNT-driven intestinal stem cell proliferation and colorectal cancer initiation. Cell Stem Cell.

[38] Wang X, Eichhorn PJA, Thiery JP (2023). TGF-beta, EMT, and resistance to anti-cancer treatment. Semin Cancer Biol.

[39] de Conti A, Tryndyak V, Heidor R, Jimenez L, Moreno FS, Beland FA (2020). Butyrate-containing structured lipids inhibit RAC1 and epithelial-to-mesenchymal transition markers: a chemopreventive mechanism against hepatocarcinogenesis. J Nutr Biochem.

[40] Lionarons DA, Hancock DC, Rana S, East P, Moore C, Murillo MM (2019). RAC1(P29S) Induces a Mesenchymal Phenotypic Switch via Serum Response Factor to Promote Melanoma Development and Therapy Resistance. Cancer Cell.

[41] Huang Y, Hong W, Wei X (2022). The molecular mechanisms and therapeutic strategies of EMT in tumor progression and metastasis. J Hematol Oncol.

[42] Hashemi M, Hajimazdarany S, Mohan CD, Mohammadi M, Rezaei S, Olyaee Y (2022). Long non-coding RNA/epithelial-mesenchymal transition axis in human cancers: Tumorigenesis, chemoresistance, and radioresistance. Pharmacol Res.

[43] Shibue T, Weinberg RA (2017). EMT, CSCs, and drug resistance: the mechanistic link and clinical implications. Nat Rev Clin Oncol.

[44] Kim TW, Kim YJ, Kim HT, Park SR, Jung JY (2016). beta-Lapachone enhances Mre11-Rad50-Nbs1 complex expression in cisplatin-induced nephrotoxicity. Pharmacol Rep.

[45] Li Y, Feng M, Guo T, Wang Z, Zhao Y (2023). Tailored Beta-Lapachone Nanomedicines for Cancer-Specific Therapy. Adv Healthc Mater.

[46] Ferraz da Costa DC, Pereira Rangel L, Martins-Dinis M, Ferretti G, Ferreira VF, Silva JL (2020). Anticancer Potential of Resveratrol, beta-Lapachone and Their Analogues. Molecules.

[47] Morris VK, Kennedy EB, Baxter NN, Benson AB 3rd, Cercek A, Cho M (2023). Treatment of Metastatic Colorectal Cancer: ASCO Guideline. J Clin Oncol.

[48] Singh M, Morris VK, Bandey IN, Hong DS, Kopetz S (2024). Advancements in combining targeted therapy and immunotherapy for colorectal cancer. Trends Cancer.

[49] Gunjur A (2019). Targeted therapy for BRAF-mutant colorectal cancer. Lancet Oncol.

[50] Chalabi M, Verschoor YL, Tan PB, Balduzzi S, Van Lent AU, Grootscholten C (2024). Neoadjuvant Immunotherapy in Locally Advanced Mismatch Repair-Deficient Colon Cancer. N Engl J Med.

[51] Williams CJM, Peddle AM, Kasi PM, Seligmann JF, Roxburgh CS, Middleton GW (2024). Neoadjuvant immunotherapy for dMMR and pMMR colorectal cancers: therapeutic strategies and putative biomarkers of response. Nat Rev Clin Oncol.

[52] Xia F, Wang Y, Wang H, Shen L, Xiang Z, Zhao Y (2024). Randomized Phase II Trial of Immunotherapy-Based Total Neoadjuvant Therapy for Proficient Mismatch Repair or Microsatellite Stable Locally Advanced Rectal Cancer (TORCH). J Clin Oncol.

[53] Gomes CL, de Albuquerque Wanderley Sales V, Gomes de Melo C, Ferreira da Silva RM, Vicente Nishimura RH, Rolim LA (2021). Beta-lapachone: Natural occurrence, physicochemical properties, biological activities, toxicity and synthesis. Phytochemistry.

