# Supplementary material for: Targeting the ARL4C/RAP1/PI3K-Akt-mTOR signaling loop promotes ARL4C ubiquitination and reverses oxaliplatin resistance in colorectal cancer
Source: Theranostics. 2026 Jan 1;16(1):99–122. doi: 10.7150/thno.117590 (PMC12665106; doi:10.7150/thno.117590)
Supplement: Supplementary file 1 — Supplementary materials and methods, figures and tables. [file thnov16p0099s1.pdf]

Supplementary materials for

Targeting the ARL4C/RAP1/PI3K-Akt-mTOR signaling loop promotes ARL4C ubiquitination and reverses oxaliplatin resistance in colorectal cancer

Yang Wang[2,3#], Zewen Chang[1,3#], Ziquan Sun[1,3#], Lu Wang[3], Jingtao Li[1,3], Guodong Li[1,3], Yuliuming Wang[2,3], Jinning Zhang[2,3], Lianjie Ai[2,3], Zhongxu Zhang[1,3], Jiaojiao Dong[1,3], and Ming Liu[1,3*]

***Correspondence:** Dr. Ming Liu (mingliu35@hrbmu.edu.cn)

This PDF file includes:

Materials and Methods

Figures S1 to S10

Tables S1 to S5

Materials and Methods

Clinical Samples and Data

Colorectal cancer (CRC) tissues, adjacent non-tumorous tissues, and normal tissues were obtained from 112 CRC patients diagnosed at the Fourth Affiliated Hospital and the Second Affiliated Hospital of Harbin Medical University. Written informed consent was obtained from all patients prior to surgical resection. Immediately after resection, tumor tissues were dissected under sterile conditions, washed with PBS to remove residual blood and contaminants, and extraneous tissues (such as blood vessels, adipose tissue, and connective tissue) were carefully removed. The tissues were then rinsed, dried using clean, dust-free absorbent paper, and cut into small pieces of approximately 1 cm^3 with sterile scissors or a scalpel. Tissue samples were rapidly frozen in liquid nitrogen for 5 minutes and subsequently stored at −80 °C to preserve RNA and protein integrity before being transported on dry ice. Clinical information, including demographics (e.g., sex, age, diabetes, hypertension), tumor factors (e.g., tumor size, pathological grade, TNM stage, mismatch repair status), clinical stage, and treatment history, was meticulously recorded. All experimental protocols were approved by the institutional ethics committees (Approval No. 2024-DWSYLLCZ-18).

Cell Culture

Human CRC cell lines (DLD-1, LOVO, HT-29, SW620, SW480, HCT-116, NCM460, CT26, MC38) and human embryonic kidney HEK293T cells were purchased from Procell Life Science & Technology Co., Ltd. (Wuhan, China). All cell lines were authenticated by short tandem repeat (STR) profiling to ensure authenticity and minimize misidentification. Cells were cultured in RPMI-1640 or DMEM medium (Gibco, New York, USA), supplemented with 10% fetal bovine serum (FBS, Gibco, Life Technologies, 10270) and 1% penicillin-streptomycin, in a humidified incubator at 37 °C with 5% CO_2. Cells were passaged every two days using 0.25% trypsin-EDTA solution.

Plasmids and Reagents

ARL4C-related plasmids, including pLKD-shRNA-ARL4C, PReceiver-ARL4C-1v105, and PReceiver-lv105 for knockdown and overexpression, were obtained from Shanghai Genechem Co., Ltd. (Shanghai, China). The shRNA sequences were as follows: shARL4C#1: GATGATCCTGAAACGCAGGAA; shARL4C#2: GCTCAAGTTCAACGAGTTCGT; shARL4C#3: CTCCTCTAACATCTCGGCCTT. For lentiviral packaging and stable transduction, HEK293T cells were co-transfected with HIV packaging plasmids and HIV lentiviral expression plasmids using the GeneCopoeia Lenti-Pac™ Lentiviral Packaging Kit (Cat. No. LT001). After 48 hours of incubation, virus-containing supernatants were collected, centrifuged at 2000 rpm for 10 minutes, and filtered through a 0.45 μm filter to remove cell debris. Based on ARL4C expression profiles, DLD-1 and LOVO cell lines were selected for knockdown construction, and HCT-116 for overexpression construction. Cells were infected with lentivirus for 72 hours and subsequently selected using puromycin. The puromycin concentrations were 2 μg/mL for LOVO, DLD-1, and HCT-116 cells.

Quantitative Real-Time PCR (qRT-PCR)

Total RNA was extracted from cells or tissue samples using TRIzol reagent (Thermo Fisher Scientific), and RNA concentration and purity were assessed using a NanoDrop spectrophotometer (Bio-Rad), ensuring a 260/280 ratio between 1.8 and 2.1. Reverse transcription was performed

using the iScript cDNA Synthesis Kit (Bio-Rad) with 1 μg of total RNA. qRT-PCR was conducted in a 20 μL reaction system containing 10 μL SYBR Green PCR Master Mix, 1 μL forward and reverse primers (10 μM), 2 μL template cDNA, and RNase-free water. The thermal cycling conditions were 95 °C for 10 minutes, followed by 40–45 cycles of 95 °C for 15 seconds and 60 °C for 1 minute. All reactions were performed in triplicate with GAPDH used as the internal control. Relative expression levels were calculated using the 2^-ΔCT method. Gradient-diluted standards were included to construct standard curves. Primer sequences used were: GAPDH: 5'-GTCTCCTCTGACTTCAACAGCG-3' (forward) and 5'-ACCACCCTGTTGCTGTAGCCAA-3' (reverse); ARL4C: 5'-GGGCAACATCTCCTCTAACAT-3' (forward) and 5'-GTTGAACTTGAGCCGGTAGA-3' (reverse).

MTT Assay

Cells were seeded at 2000 cells/well in 96-well plates. To assess the effects of oxaliplatin on cell viability, KD-ARL4C, NC, OE-ARL4C, and Vector CRC cells were treated with various concentrations of oxaliplatin (0, 0.5, 1, 2.5, 5, 7.5, 10, 15, 20 μM) for 24 or 48 hours. Subsequently, 15 μL of 5 mg/mL MTT solution (Sigma-Aldrich, MO, USA) was added to each well and incubated for 4 hours. After removing the medium, 100 μL DMSO was added to dissolve the formazan crystals. Absorbance was measured at 570 nm with a reference at 630 nm using a microplate reader. Each experiment was performed in triplicate.

Coefficient of Drug Interaction (CDI)

The CDI was used to quantitatively evaluate drug interaction effects. It was calculated as CDI = E_AB / (E_A × E_B), where E_AB represents the relative effect of combined treatment, and E_A and E_B represent the effects of single agents. CDI values were interpreted as follows: CDI < 1 indicates synergism; CDI = 1 indicates an additive effect; and CDI > 1 indicates antagonism.

Transwell Invasion Assay

Cell invasion was assessed using Transwell chambers (Corning, New York, USA) with an 8 μm pore membrane pre-coated with Matrigel (BD Biosciences, San Jose, USA) diluted 1:8 and solidified at 37 °C for 1 hour. Cells were suspended in serum-free medium at 1×10^5 cells/100 μL and seeded into the upper chamber. The lower chamber contained 600 μL medium with 10% FBS. After 24–48 hours of incubation at 37 °C with 5% CO_2, non-invading cells were removed, and invaded cells were fixed with 4% paraformaldehyde, stained with 0.1% crystal violet, washed, and counted under an inverted microscope (×200 magnification) in at least five fields per sample. ImageJ software was used for quantification.

Wound Healing Assay

To assess the role of ARL4C in CRC cell migration, stable ARL4C knockdown and overexpression cell lines were generated. After transfection, cells were seeded into 6-well plates and grown to approximately 80% confluence. A scratch was made using a sterile 200 μL pipette tip. Detached cells were removed by washing with PBS, and cells were cultured in serum-free DMEM with or without 10 μM oxaliplatin. Images were captured at 0, 24, and 48 hours to monitor wound closure. Each experiment was independently repeated at least three times.

EdU Assay

Cells were seeded into 6-well plates and cultured to approximately 70% confluence. Cells were then incubated with 10 μM EdU (RiboBio EdU Apollo567 Kit) for 2 hours. After fixation with 4% paraformaldehyde and permeabilization with 0.3% Triton X-100, EdU staining was

performed following the manufacturer’s protocol. Nuclei were counterstained with Hoechst 33342. Fluorescent images were captured, and EdU-positive cells were quantified. All experiments were repeated at least three times.

Live/Dead Cell Staining Assay

Cells from KD-ARL4C (DLD-1, LOVO) and OE-ARL4C (HCT-116) groups, along with corresponding oxaliplatin-treated groups, were seeded in multi-well plates. After treatment, cells were washed with PBS and stained with Calcein-AM/PI working solution (100 µL per well) at 37 °C for 30 minutes in the dark. Calcein-AM labeled live cells with green fluorescence (Ex/Em = 494/517 nm), and PI stained dead cells with red fluorescence (Ex/Em = 535/617 nm). Fluorescence images were captured using a fluorescence microscope (N2-DMi8, Leica).

Western Blotting

Total proteins were extracted using RIPA buffer supplemented with protease and phosphatase inhibitors (Thermo Fisher Scientific). Protein concentrations were determined using a BCA assay (Thermo Scientific). Equal amounts of protein were separated by SDS-PAGE and transferred onto PVDF membranes (0.45 µm, Millipore, USA). Membranes were incubated overnight at 4 °C with primary antibodies against various targets (listed in detail above), followed by HRP-conjugated secondary antibodies (1:5000 dilution) for 1 hour. Signal detection was performed using an enhanced chemiluminescence substrate (Thermo Fisher Scientific) and visualized with a ChemiDoc MP Imaging System (Bio-Rad). GAPDH was used as a loading control. Densitometric analysis was conducted using ImageJ software (v1.48, NIH, USA). Primary antibodies used for this experiment were as follows: ARL4C (AB122025-1001, Abcam), RAP1A(Cat No : 4938, CST), RAC1(Cat No : 4651, CST), Bax(Cat No. 60267-1-Ig, Proteintech), Bcl2(Cat No : 15071, CST), Cleaved caspase-3(Cat No : 9664, CST), Cleaved caspase-8(Cat No : 8592, CST), Cleaved caspase-9(Cat No : 7237, CST), Caspase-3(Cat No. 19677-1-AP, Proteintech), Caspase-8(Cat No. 66093-1-Ig, Proteintech), Caspase-9(Cat No. 10380-1-AP, Proteintech), E-cad(Cat No : 3195, CST), Vimentin(Cat No. 60330-1-Ig, Proteintech), N-cad(Cat No. 22018-1-AP, Proteintech), RAP1GAP(Cat No. MA5-42831, Thermo Scientific), RAP1(Cat No. MA5-17164, Thermo Scientific), P-AKTS473 (Cat No : 4060, CST), AKT(Cat No. 60203-2-Ig, Proteintech), p-PI3K (AP0854, Abclonal), PI3K(A19742, Abclonal), P-mTOR(Cat No : 5536,CST), mTOR(Cat No : 2983, CST), Arp2/3(Cat No. A7767, Abclonal) Ubiquitin (Cat No : 58395, CST) and GAPDH (Cat No. 60004-1-Ig, Proteintech).

Co-Immunoprecipitation (Co-IP)

ARL4C protein was extracted from OE-ARL4C HCT-116 cells. Cells were washed with cold PBS, lysed with buffer containing PMSF and protease inhibitors, and centrifuged to collect the supernatant for Co-IP assays. The supernatant was incubated with ARL4C antibody (Cat No. 10202-1-AP, Proteintech) or control IgG overnight at 4°C, followed by incubation with Protein G magnetic beads. After washing, the bound proteins were eluted and analyzed by Western blot. For SDS-PAGE, silver staining was performed: gels were fixed, washed, stained, and rinsed to visualize protein bands.

Mass Spectrometry Analysis

Mass spectrometry analysis was performed by Hangzhou Jingjie Biotechnology Co., Ltd. ARL4C-enriched proteins from OE-ARL4C HCT-116 cells were subjected to SDS-PAGE, with approximately 20 µL of protein loaded per gel. After electrophoresis, protein bands were excised, washed, and cut into 1 mm^3 pieces. These were treated with DTT and IAA for reduction and

alkylation, followed by dehydration and digestion with trypsin. Peptides were extracted sequentially with acetonitrile solutions and lyophilized for mass spectrometric analysis. Raw MS data were processed using Proteome Discoverer or MaxQuant for protein identification and quantification.

Immunofluorescence (IF)

The subcellular localization of ARL4C, RAP1A, and RAC1 was examined in colorectal cancer cells. Cells were cultured on coverslips, fixed with 4% paraformaldehyde (15 min, room temperature), permeabilized with 0.1% Triton X-100 (10 min), and blocked with 1% BSA or serum (30–60 min). Cells were incubated overnight at 4°C with the following primary antibodies: ARL4C (Cat No. AB122025-1001, Abcam), RAC1 (Cat No. 66122-1-Ig, Proteintech), RAP1A (Cat No. 66122-1-Ig, Proteintech), p-AKT^{S473} (Cat No: 23430, CST), AKT (Cat No: 9272, CST), p-mTOR (Cat No: 5536, CST), and mTOR (Cat No. 66888-1-Ig, Proteintech). After washing with PBST (PBS with Tween-20), cells were incubated with Alexa Fluor 488- or Alexa Fluor 594-conjugated secondary antibodies (Abcam) at room temperature for 1 hour in the dark. Nuclei were counterstained with DAPI (Thermo Fisher Scientific) for 5 minutes. Coverslips were mounted using antifade mounting medium (Beyotime). Confocal images were acquired using a Nikon C2+ confocal microscope equipped with standard filters and NIS-Elements software to determine the subcellular distribution of target proteins based on multichannel fluorescence signals.

Molecular Docking

Molecular docking was performed to validate potential interactions between proteins or between proteins and small molecules. The HDOCK web server (http://hdock.phys.hust.edu.cn/) was employed for docking simulations. HDOCK evaluates protein-protein docking conformations, binding affinities, and interacting residues within 5 Å. Structural models of docking partners were retrieved from the AlphaFold and Protein Data Bank (PDB) databases, including ARL4C (ID: P56559), USP1 (ID: O94782), USP35 (ID: Q9P2H5), USP37 (ID: Q86T82), USP38 (ID: Q8NB14), USP42 (ID: Q9H9J4), and USP49 (ID: Q70CQ1). Docking was performed between ARL4C and each of RAP1A, RAC1, the aforementioned USPs, and the small molecule β-Lapachone. LigPlus software was used to analyze 2D interaction forces, while PyMOL (version 2.6.0) was employed to visualize amino acid residues involved in interactions. Detailed docking data are provided in Table S5 (Supporting Information).

Animal Models

Male BALB/c nude and BALB/c mice (5–6 weeks old) were obtained from Charles River (Beijing, China). All animals were maintained under specific pathogen-free (SPF) conditions at the Animal Facility of the Fourth Affiliated Hospital of Harbin Medical University. All experimental procedures were approved by the Institutional Animal Care and Use Committee (IACUC) of the same institution (Approval No. 2024-DWSYLLCZ-18).

Subcutaneous Tumor Model

For BALB/c nude mice, OE-ARL4C or vector-transfected HCT-116 cells (5×10^6 cells in 0.1 mL PBS) were subcutaneously injected into the left shoulder. Mice received intraperitoneal injections of oxaliplatin (10 mg/kg/day). Mice were randomly divided into four groups (n = 7 per group): vector, oxaliplatin, OE-ARL4C, and OE-ARL4C + oxaliplatin. For BALB/c mice, CT26 cells (1×10^6 cells in 0.1 mL PBS) were injected subcutaneously. Mice received intraperitoneal injections of oxaliplatin and/or β-Lapachone at 10 mg/kg/day. Animals were randomly divided

into four groups (n = 7 per group): vehicle, oxaliplatin, β-Lapachone, and oxaliplatin + β-Lapachone. Tumor volume was measured every 3 days using calipers once it reached 100 mm^3. Tumor volume was calculated using the formula: Volume = length × $(depth)^2$ × π/6. After three weeks, mice were euthanized, tumors were excised, weighed, and fixed in 10% neutral-buffered formalin for histological analysis.

Orthotopic Colorectal Cancer Mouse Model

Mice were anesthetized with 3% isoflurane, and abdominal hair was removed. A 1 – 2 cm midline incision was made to expose the abdominal cavity. The mesenteric triangle of the cecum was selected for cell injection due to its rich blood supply and minimal obstruction risk. Using a 26G or 27G needle, 100 μL of CT26 (1×10^6 cells), KD-ARL4C-DLD-1 (5×10^6 cells), or NC/Control cells were injected into the mesenteric triangle of BALB/c or BALB/c Nude mice. After injection, the incision was closed, and the mice recovered in a warm environment. Tumor growth was monitored by MRI, and tissues were collected for H&E staining. This model effectively mimics human colorectal cancer progression, inducing metastasis to the liver, spleen, diaphragm, and lymph nodes.

Metastasis Models

Pulmonary metastasis models were established by tail vein injection of CRC cells (KD-ARL4C-DLD-1 or NC: 5×10^6 cells/100 μL PBS; CT26: 1×10^6 cells/100 μL PBS) into 5-week-old BALB/c Nude or BALB/c mice. Tumor development and lung metastasis were monitored by MRI. Upon visible nodule formation, mice were euthanized, and lungs were collected for metastatic nodule counting, weighing, and histological analysis. For liver metastasis, anesthetized mice underwent left subcostal laparotomy. CRC cells (same concentrations as above) were injected into the splenic parenchyma. After ensuring no leakage, the abdomen was sutured, and mice recovered on a 37°C heating pad. MRI was used to monitor tumor growth. Mice were treated with oxaliplatin (10 mg/kg/day) and/or β-Lapachone to assess therapeutic effects. Upon detection of hepatic metastases, mice were sacrificed, and liver tissues were analyzed by nodule counting, weighing, H&E staining, and histology. Grouping was consistent across models.

Hematoxylin and Eosin (H&E) Staining

Tumor tissues were fixed in 4% paraformaldehyde for 24 hours, dehydrated through graded ethanol, and embedded in paraffin. Sections (4 – 6 μm thick) were cut and incubated at 62°C for 24 hours to enhance adhesion. After dewaxing with xylene I and II, sections were rehydrated through descending ethanol concentrations (100%, 95%, and 80%), washed thoroughly in distilled water, and stained with hematoxylin for 5 – 10 minutes to visualize nuclear structures. Non-specific staining was removed by differentiation with 1% hydrochloric acid ethanol, followed by bluing under running water. Eosin staining (0.5 – 5%) was performed to highlight the cytoplasm and extracellular matrix. After staining, sections were washed with distilled water, dehydrated through graded ethanol, cleared with xylene, and mounted with neutral resin.

Immunohistochemistry (IHC)

Paraffin-embedded sections (4 μm thick) were dewaxed with xylene, rehydrated through an ethanol gradient, and subjected to antigen retrieval in citrate buffer using microwave pressure treatment for 6 minutes. Sections were cooled at room temperature for 30 minutes, endogenous peroxidase was blocked with 3% hydrogen peroxide in methanol for 30 minutes, and non-specific binding was blocked with 5% goat serum. Primary antibodies, including ARL4C (AB122025-1001, Abcam), RAC1 (Cat No. 66122-1-Ig, Proteintech), Cleaved caspase-3 (Cat No:

9664, CST), N-cadherin (Cat No. 22018-1-AP, Proteintech), and Ki-67 (Cat No. 27309-1-AP, Proteintech), were diluted in PBS/0.1% Tween-20 and incubated overnight at 4° C. The following day, sections were incubated with the secondary antibody using the VECTASTAIN Elite ABC Kit, visualized with DAB, counterstained with hematoxylin, dehydrated through graded ethanol, cleared with xylene, and mounted with neutral resin. Semi-quantitative scoring was performed independently by three blinded pathologists based on staining intensity and distribution for subsequent statistical analysis.

Bioinformatics Analysis

Bioinformatic analyses were conducted to investigate colorectal cancer (COADREAD)-associated datasets. Differentially expressed proteins (DEPs) from DIA proteomics and mass spectrometry results were identified based on fold change (>1.2 or <0.83) and p-value <0.05. Quality control, background correction, and normalization were performed prior to analysis. Gene Ontology (GO) annotation (http://geneontology.org/; http://www.blast2go.com/b2ghome) and Kyoto Encyclopedia of Genes and Genomes (KEGG) pathway analysis (http://www.genome.jp/kegg/) were used to annotate and enrich identified DEPs. Protein-protein interaction networks were constructed using the STRING database (https://string-db.org/). ARL4C expression levels were compared between colorectal cancer and normal controls using UALCAN (http://ualcan.path.uab.edu/) and Sangerbox (http://sangerbox.com/). The TNMplot database (https://tnmplot.com/Analysis/) was utilized to examine ARL4C expression in normal, tumor, and metastatic tissues. Expression of ARL4C across different histological subtypes, lymph node stages, and mutation conditions was also evaluated via UALCAN. Protein expression profiles of ARL4C and related molecules were obtained from the CPTAC database (https://cptac-data-portal.georgetown.edu/cptac/). Correlation analyses were performed using TIMER2.0 (http://timer.comp-genomics.org/) and Linkedomics (http://www.linkedomics.org/login.php). Prognostic relevance of ARL4C was assessed using TIMER2.0 and survival analyses (OS, RFS, PFS, and PPS) were conducted via KMPLOT (http://kmplot.com/analysis/).

Statistical Analysis

Statistical analyses were performed using SPSS 26.0 (IBM, New York, USA) and GraphPad Prism 9.5 (GraphPad Software, San Diego, CA, USA). Data following a normal distribution were presented as mean ± standard deviation (SD) and analyzed using independent-sample t-tests or one-way ANOVA with Bonferroni or Tamhane's post hoc tests. The Bonferroni method adjusts the significance threshold (α) by dividing the standard 0.05 level by the number of comparisons. This ensures that the family-wise error rate is controlled. Non-normally distributed data were analyzed using the Mann-Whitney U test or Kruskal-Wallis test. Paired t-tests were used for comparisons of protein expression levels, and Spearman's rank correlation coefficient was employed for correlation analyses. Kaplan-Meier survival curves were constructed, and differences were assessed by log-rank tests; Cox proportional hazards models were used for multivariate analyses. The association between ARL4C expression and clinicopathological features was evaluated using the Chi-square test. *$p < 0.05$, **$p < 0.01$, and ***$p < 0.001$ were considered statistically significant.

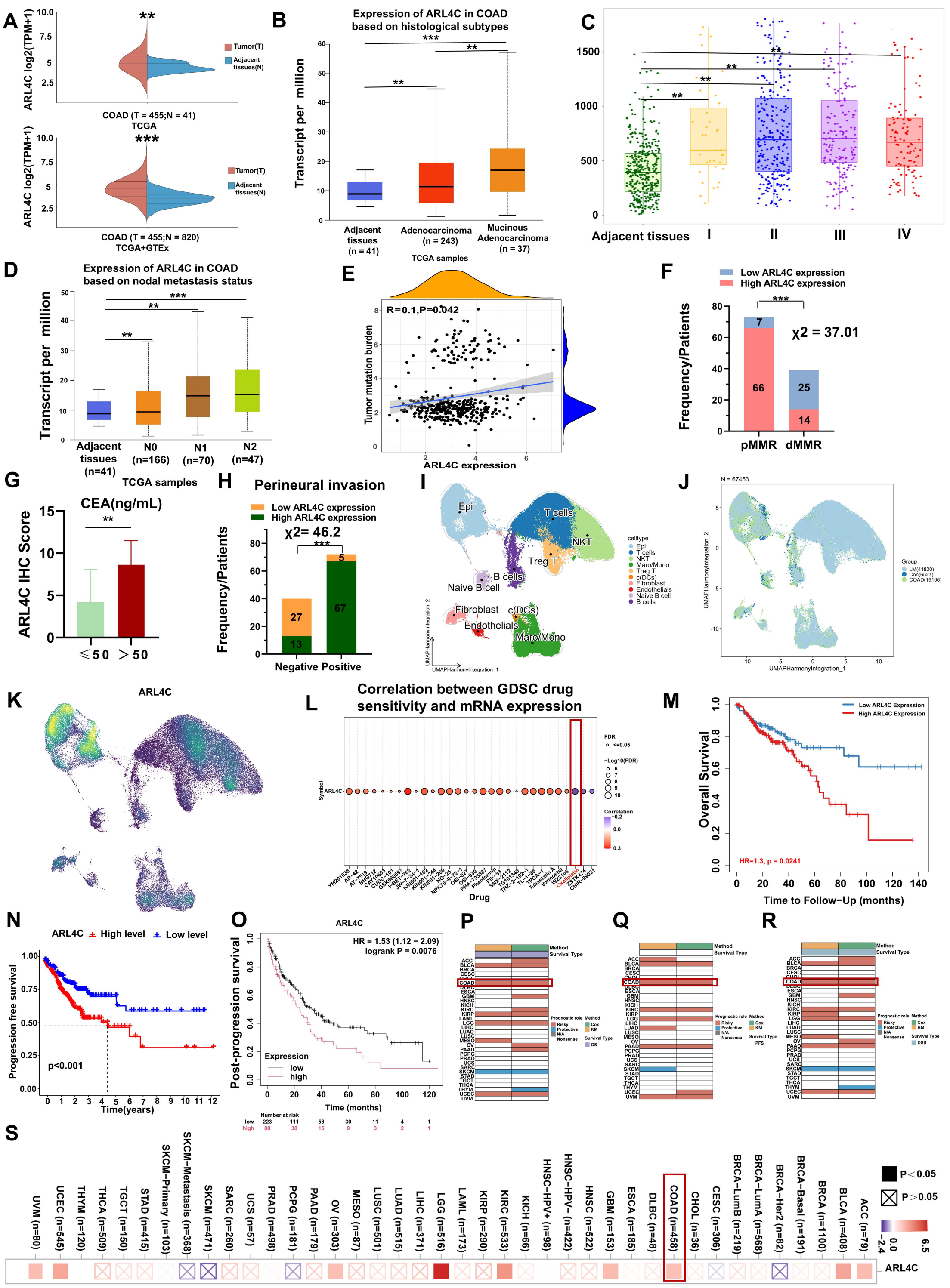

Figure S1. (A) Expression levels of ARL4C in colorectal cancer and normal tissues were analyzed using TCGA or TCGA+GTEx datasets. **(B)** ARL4C expression in normal tissues, adenocarcinoma, and mucinous adenocarcinoma was analyzed using TCGA data. **(C)** The TNMplot database was used to examine ARL4C expression across normal tissues and different tumor stages in colorectal cancer. **(D)** ARL4C expression in relation to lymph node stages of colorectal cancer was evaluated via the UALCAN database. **(E)** Correlation analysis between ARL4C expression and tumor mutation burden (TMB) in TCGA dataset. **(F)** Distribution of ARL4C expression in pMMR and

dMMR colorectal cancer patients. Patients were stratified by ARL4C expression (high vs. low) and MMR status (pMMR vs. dMMR). Statistical significance was assessed using the chi-square test. ***P < 0.001. **(G)** Correlation between ARL4C expression and CEA levels (clinical samples). **(H)** Correlation between ARL4C expression and perineural invasion in colorectal cancer (clinical samples).Statistical significance was assessed using the chi-square test. ***P < 0.001. **(I-K)** UMAP visualization of the GSE231559 single-cell RNA-sequencing dataset. **(L)** Relationship between ARL4C expression and drug sensitivity across various agents in the GDSC database. **(M-O)** Prognostic impact of ARL4C expression on OS (TIMER2.0, p = 0.0241), PFS (TCGA, p < 0.001), and PPS (KM-plotter, p = 0.0076). **(P-R)** Pan-cancer risk analysis of ARL4C expression for OS, PFS, and DSS. **(S)** Correlation analysis between ARL4C and multiple cancer types using TIMER2.0. (* p < 0.05, ** p < 0.01, *** p < 0.001, ns. p > 0.05)

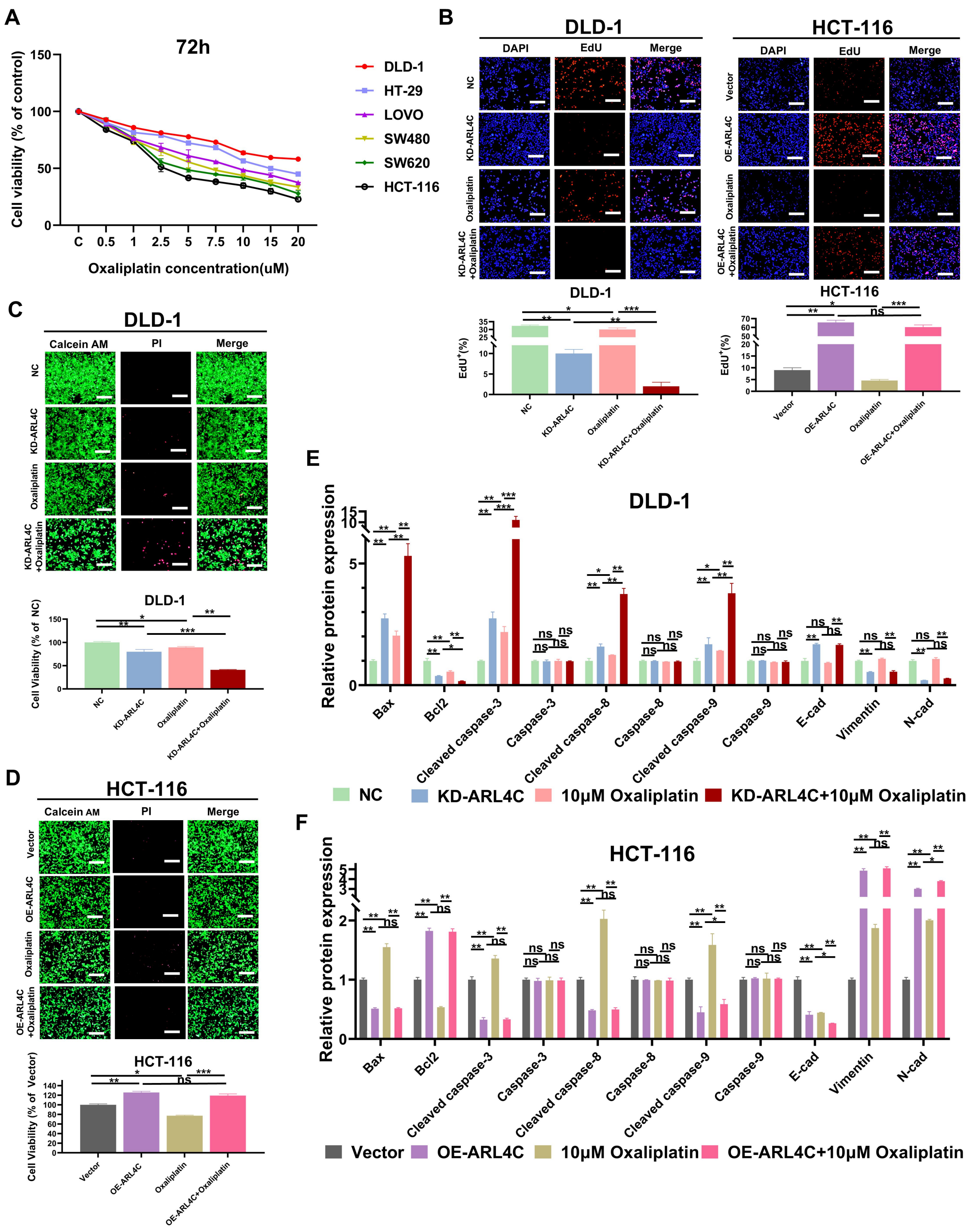

Figure S2. (A) Dose-dependent cell viability of six human colorectal cancer cell lines upon oxaliplatin treatment. **(B)** EdU assay evaluating the effect of ARL4C knockdown/overexpression and oxaliplatin on proliferation. **(C-D)** Live/dead staining for apoptosis. **(E-F)** Western blot quantification of apoptosis- and EMT-related proteins. (* p < 0.05, ** p < 0.01, *** p < 0.001, ns. p > 0.05)

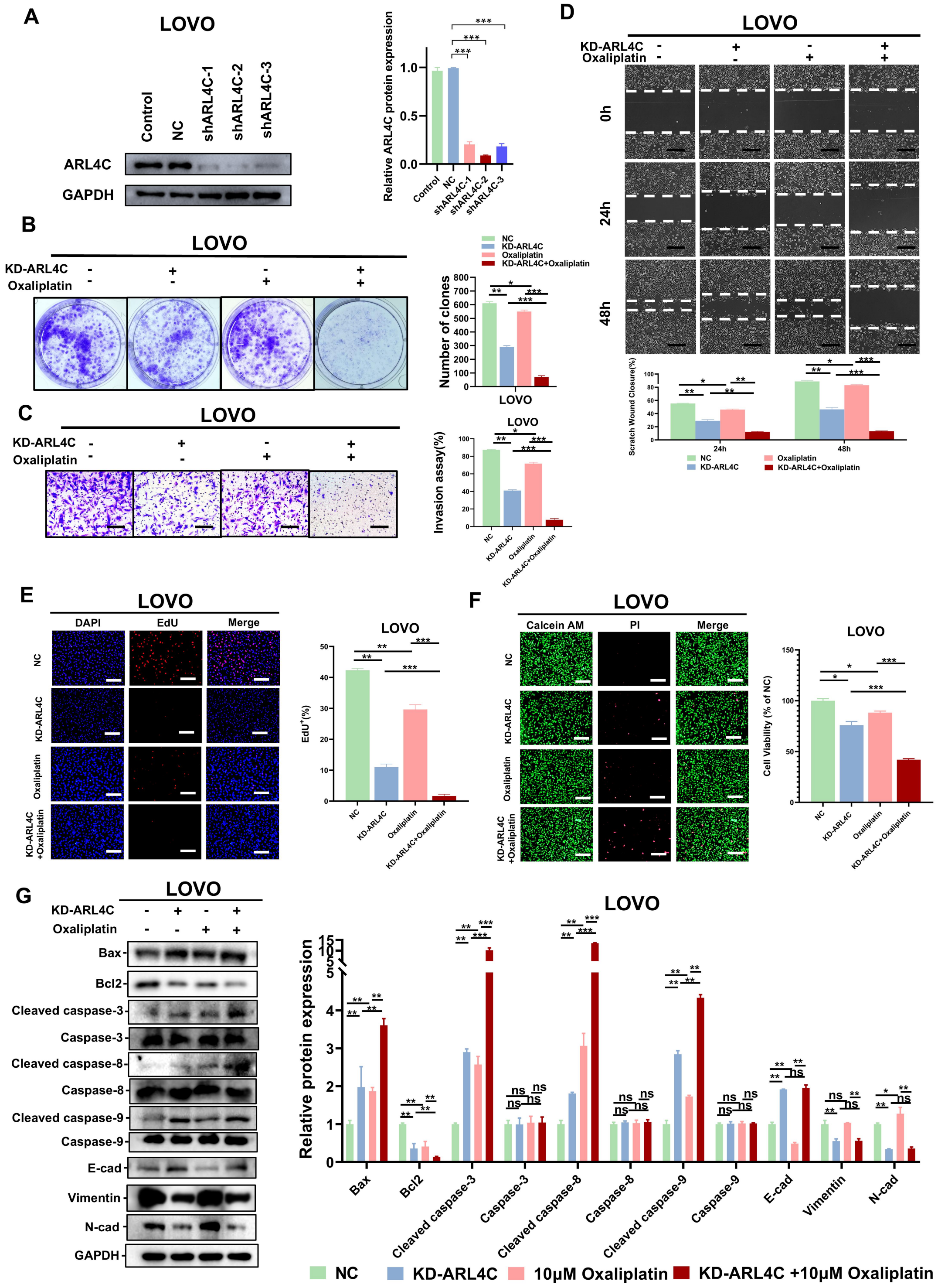

Figure S3. (A) Validation of ARL4C knockdown efficiency. **(B)** Colony formation assay. **(C)** Transwell invasion assay. **(D)** Wound healing assay. **(E)** EdU proliferation assay. **(F)** Live/dead staining. **(G)** Western blot for apoptosis and EMT proteins. (* $p < 0.05$, ** $p < 0.01$, *** $p < 0.001$, ns. $p > 0.05$)

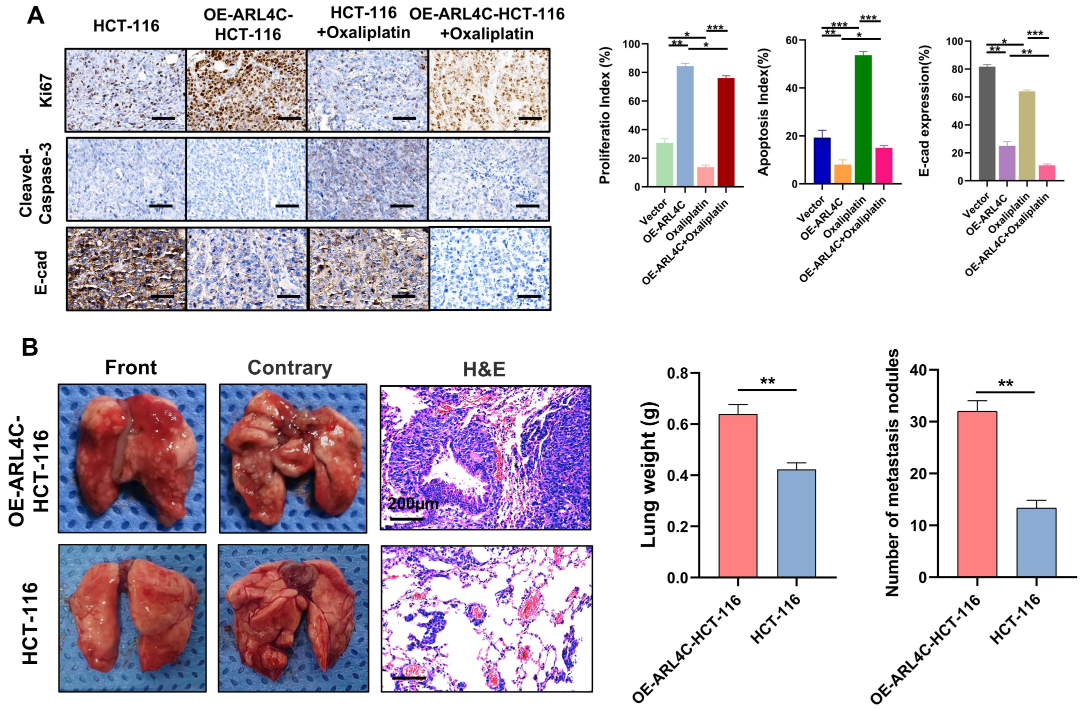

Figure S4. **(A)** Immunohistochemistry for proliferation, apoptosis, and EMT-related proteins in subcutaneous xenografts after ARL4C overexpression and oxaliplatin treatment.**(B)** A comparative analysis of lung metastasis formation between ARL4C-overexpressing and control HCT116 cell lines in xenograft models. (* $p < 0.05$, ** $p < 0.01$, *** $p < 0.001$, ns. $p > 0.05$)

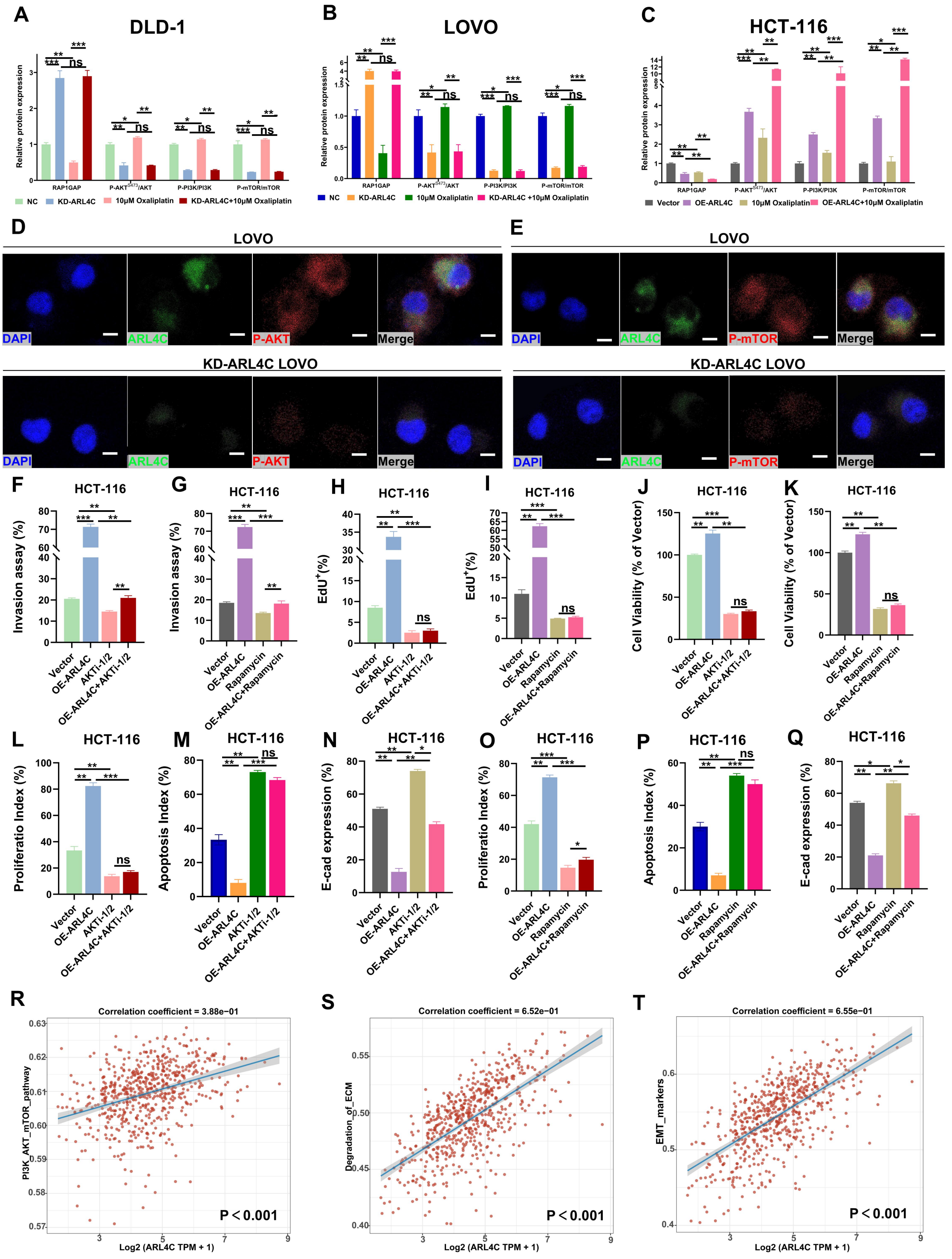

Figure S5. (A-C) Quantification of pathway-related proteins via Western blot. **(D-E)** Immunofluorescence for P-AKT and P-mTOR. **(F-G)** Transwell invasion assay with AKTi-1/2 or rapamycin. **(H-I)** EdU assay. **(J-K)** Live/dead staining. **(L-Q)** IHC of xenografts for proliferation, apoptosis, and EMT markers. **(R-T)** TCGA correlation analysis of ARL4C with PI3K-Akt/mTOR, ECM degradation, and EMT markers. (* $p < 0.05$, ** $p < 0.01$, *** $p < 0.001$, ns. $p > 0.05$)

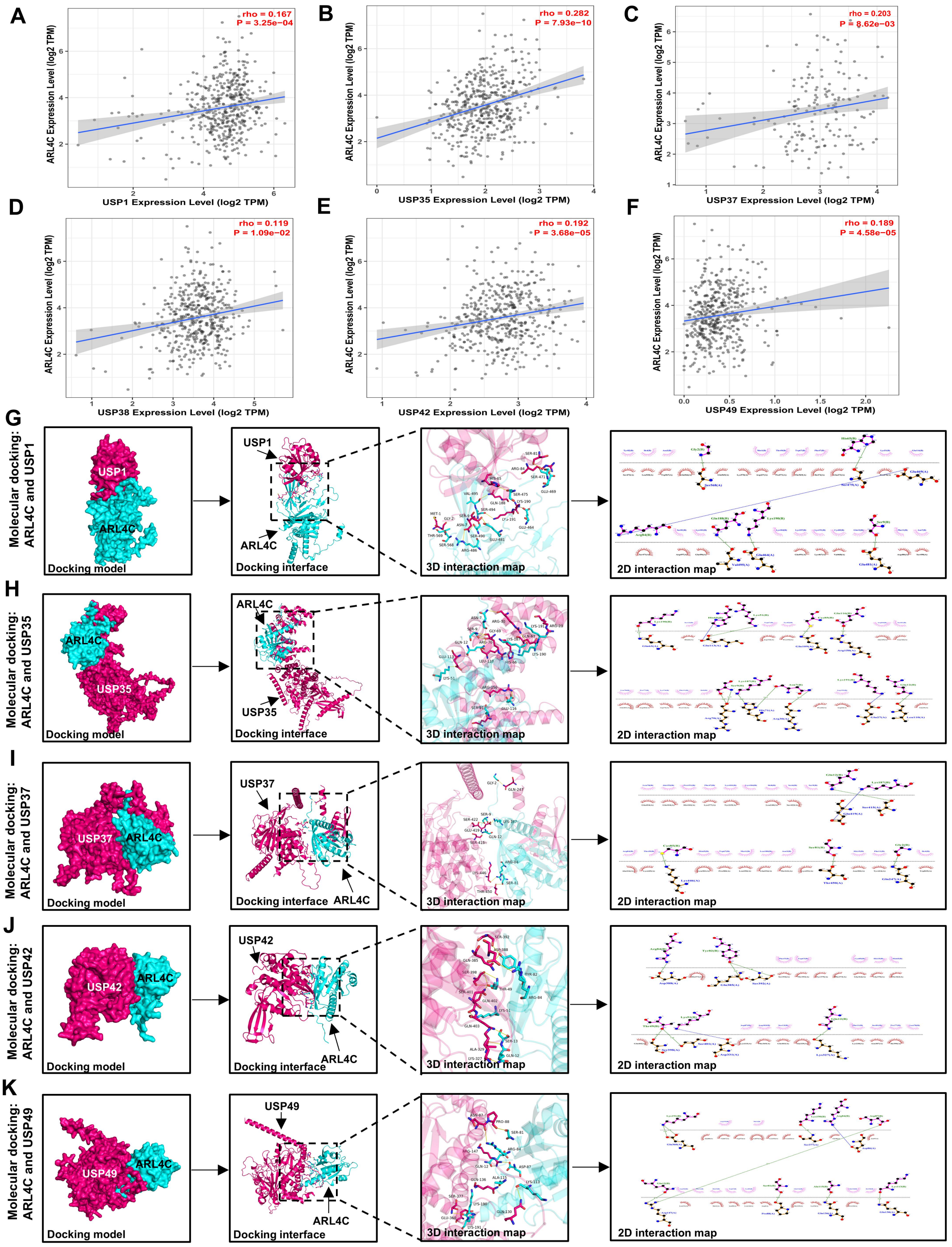

Figure S6. (A-F) Correlation between ARL4C and each DUBs (USP1, USP35, USP37, USP38, USP42, USP49). **(G-K)** Molecular docking and interaction maps of ARL4C with each DUBs. (* p < 0.05, ** p < 0.01, *** p < 0.001, ns. p > 0.05)

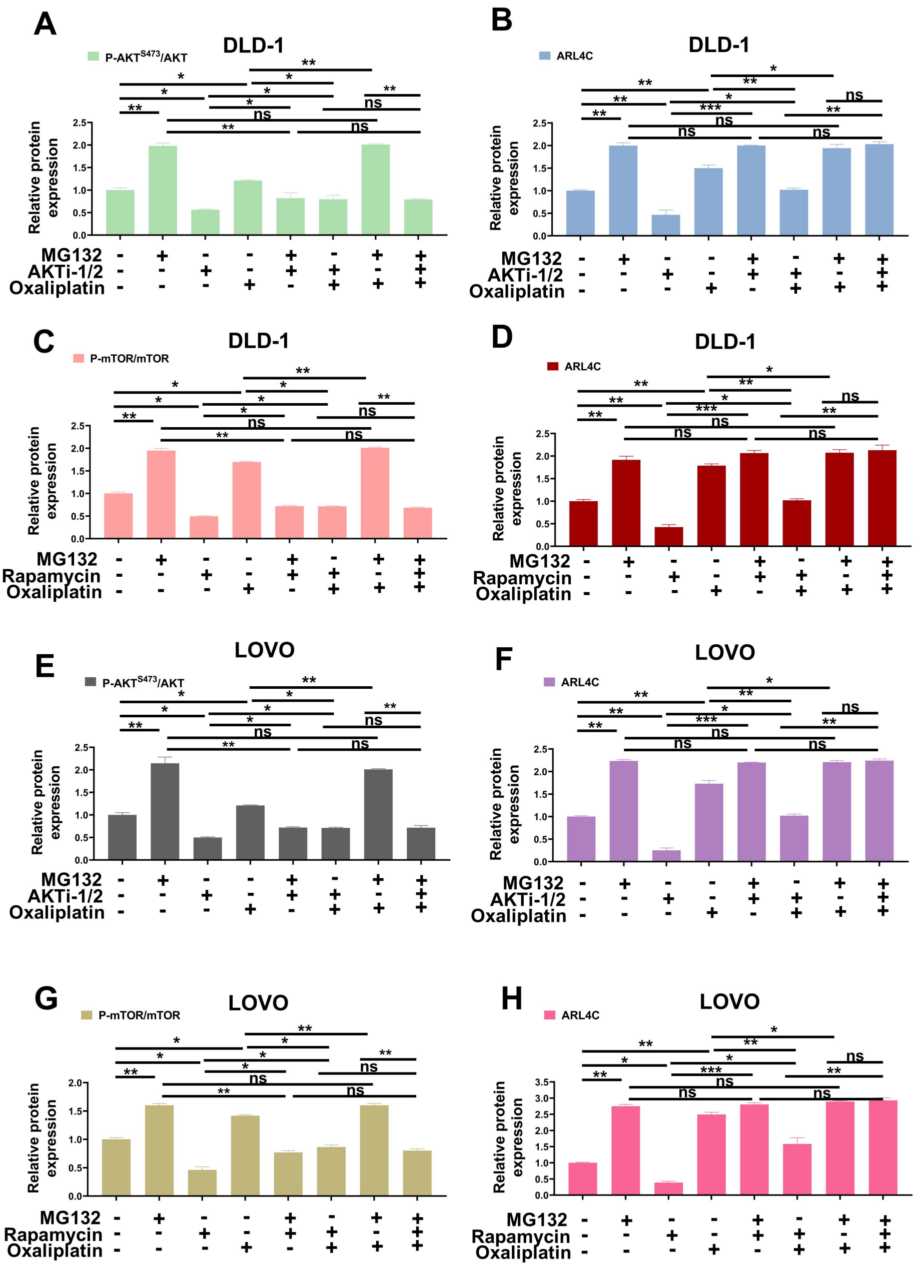

Figure S7. (A-B, E-F) Quantification of ARL4C and P-AKT^{S473} protein levels after MG132, AKTi-1/2, or oxaliplatin treatment. **(C-D, G-H)** Quantification of ARL4C and P-mTOR levels under the same treatments. (* $p < 0.05$, ** $p < 0.01$, *** $p < 0.001$, ns. $p > 0.05$)

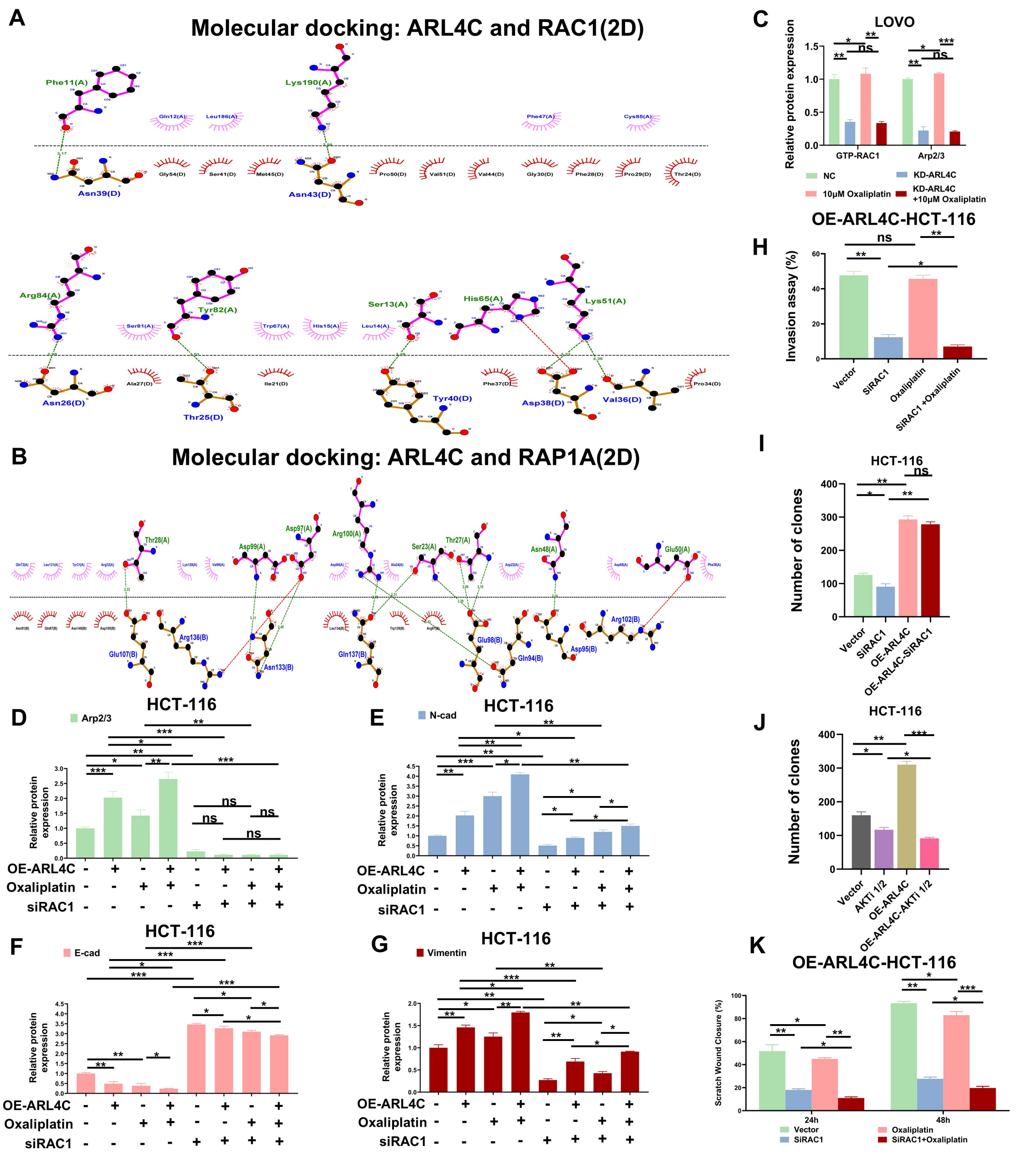

Figure S8. (A) 2D molecular docking of ARL4C with RAC1. **(B)** 2D docking of ARL4C with RAP1A. **(C)** GTP-RAC1 and Arp2/3 protein changes by Western blot. **(D-G)** Western blot of EMT and Arp2/3 proteins after ARL4C overexpression and RAC1 knockdown. **(H)** Transwell invasion assay. **(I-J)** Colony formation assay. **(K)** Wound healing assay. (* p < 0.05, ** p < 0.01, *** p < 0.001, ns. p > 0.05)

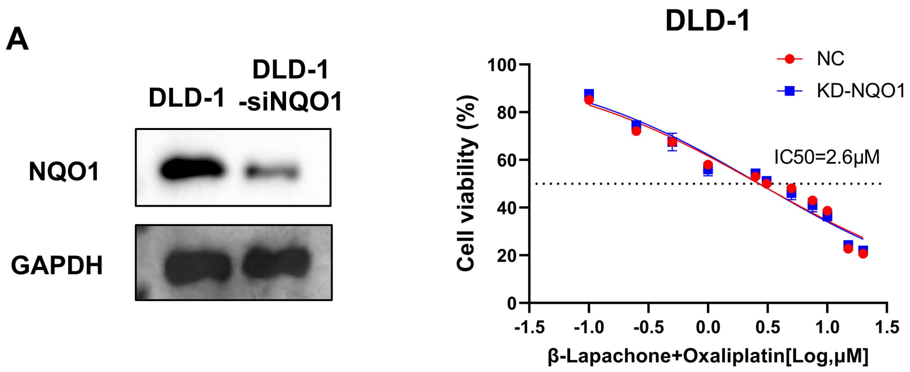

Figure S9. (A) Effect of NQO1 Knockdown on the Synergistic Action of β-Lapachone and Oxaliplatin.

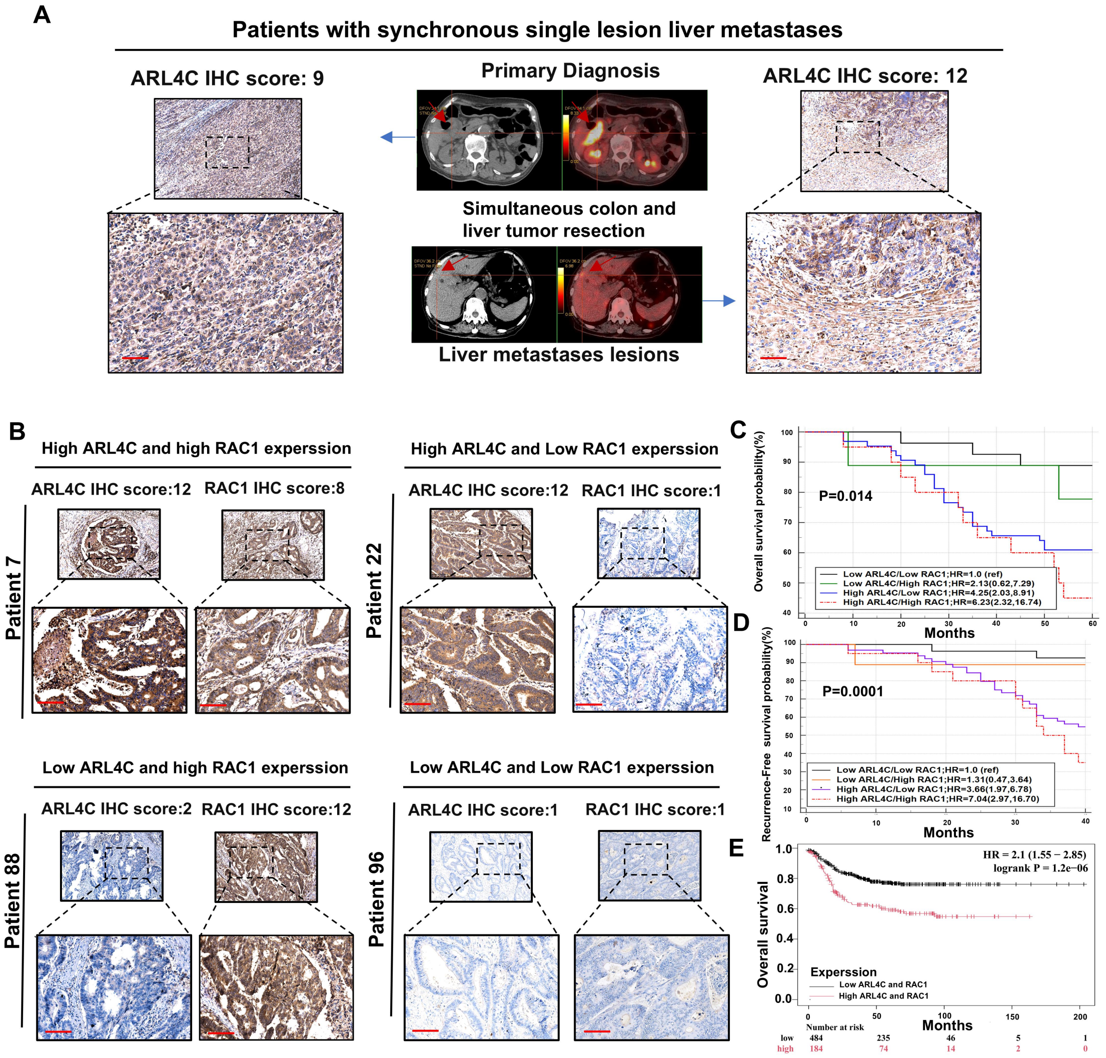

Figure S10. (A) IHC and radiologic evidence showing high ARL4C expression in synchronous liver metastases of CRC**. (B)** IHC analysis of ARL4C and RAC1 in CRC patients. **(C)** Combined ARL4C and RAC1 expression predicts OS (p = 0.014). **(D)** Combined expression predicts RFS (p = 0.0001). **(E)** OS analysis in TCGA patients with co-high or co-low expression of ARL4C and RAC1.

Table S1. Baseline characteristics of patients

Characteristics		ARL4C Expression		Total
		Low（IHC score ≤6）	High (IHC score ＞6）	112
Demographics				
Sex	Male	9(8.0%)	28(25.0%)	37(33.0%)
	Female	23(20.5%)	52(46.5%)	75(67.0%)
Age (year)	<60	13(11.6%)	21(18.8%)	34(30.4%)
	≥60	19(17.0%)	59(52.6%)	78(69.6%)
Diabetes	No	25(22.3%)	64(57.2%)	89(79.5%)
	Yes	7(6.3%)	16(14.2%)	23(20.5%)
hypertension	No	20(17.9%)	59(52.6%)	79(70.5%)
	Yes	12(10.7%)	21(18.8%)	33(29.5%)
Tumor factor				
Tumor size	<5 cm	14(12.5%)	40(35.7%)	54(48.2%)
	≥5 cm	18(16.1%)	40(35.7%)	58(51.8%)
Tumor Pathological Grade	High Differentiation	22(19.6%)	7(6.3%)	29(25.9%)
	Moderate and Low Differentiation	10(8.9%)	73(65.2%)	83(74.1%)
TNM Stage	High-risk II	24(21.4%)	14(12.5%)	38(33.9%)
	III-IV	8(7.2%)	66(58.9%)	74(66.1%)
Ki67(%)	＜20%	25(22.3%)	14(12.5%)	39(34.8%)
	≥20%	7(6.3%)	66(58.9%)	73(65.2%)
MMR	dMMR	25(22.3%)	14(12.5%)	39(34.8%)
	pMMR	7(6.3%)	66(58.9%)	73(65.2%)
Venous invasion	No	15(13.4%)	42(37.5%)	57(50.9%)
	Yes	17(15.2%)	38(33.9%)	55(49.1%)
Perineural invasion	No	27(24.1%)	13(11.6%)	40(35.7%)
	Yes	5(4.5%)	67(59.8%)	72(64.3%)
Number of lymph nodes detected	＜10	18(16.1%)	38(33.9%)	56(50.0%)
	≥10	14(12.5%)	42(37.5%)	56(50.0%)
Cancer nodules	0	17(15.2%)	18(16.1%)	35(31.3%)
	1~3	15(13.4%)	62(55.3%)	77(68.7%)
CEA（ng/mL）	≤50	21(18.8%)	18(16.1%)	39(34.9%)
	＞50	11(9.8%)	62(55.3%)	73(65.1%)
CA19-9（U/ml）	≤111	17(15.2%)	43(38.4%)	60(53.6%)
	＞111	15(13.4%)	37(33.0%)	52(46.4%)
Neutrophils（*10^9）	1.8~6.3	17(15.2%)	41(36.6%)	58(51.8%)
	＜1.8 or ＞6.3	15(13.4%)	39(34.8%)	54(48.2%)
Hemoglobin（g/L）	120~160	16(14.3%)	43(38.4%)	59(52.7%)
	＜120	16(14.3%)	37(33.0%)	53(47.3%)
lymphocyte（*10^9）	1.5-3.3	17(15.2%)	41(36.6%)	58(51.8%)
	＜1.5 or ＞3.3	15(13.4%)	39(34.8%)	54(48.2%)
Albumin（g/L）	≥35	18(16.1%)	41(36.6%)	59(52.7%)

	＜35	14(12.5%)	39(34.8%)	53(47.3%)
WBC（*10^9）	4.0～10.0	18(16.1%)	42(37.5%)	60(53.6%)
	＜4 or ＞10	14(12.5%)	38(33.9%)	52(46.4%)
PLT（*10^9）	100~300	17(15.2%)	39(34.8%)	56(50.0%)
	＜100 or ＞300	15(13.4%)	41(36.6%)	56(50.0%)
CRP	0~10	16(14.3%)	36(32.1%)	52(46.4%)
	＞10	16(14.3%)	44(39.3%)	60(53.6%)
Recurrence	No	26(23.2%)	31(27.7%)	57(50.9%)
	Yes	6(5.4%)	49(43.7%)	55(49.1%)
Vital Status	Alive	28(25.0%)	44(39.3%)	72(64.3%)
	Dead	4(3.6%)	36(32.1%)	40(35.7%)

Table S2: Association between ARL4C expression and clinicopathology of patients with colorectal carcinoma

Variables		ARL4C Expression Low（IHC Score≤6）	ARL4C Expression High（IHC Score＞6）	Total =112	χ^2	*P* value
Demographics						
Sex	Male	9(8.0%)	28(25.0%)	37(33.0%)	0.488	0.485
	Female	23(20.5%)	52(46.5%)	75(67.0%)		
Age (year)	<60	13(11.6%)	21(18.8%)	34(30.4%)	43.324	0.187
	≥60	19(17.0%)	59(52.6%)	78(69.6%)		
Diabetes	No	25(22.3%)	64(57.2%)	89(79.5%)	0.049	0.824
	Yes	7(6.3%)	16(14.2%)	23(20.5%)		
hypertension	No	20(17.9%)	59(52.6%)	79(70.5%)	1.392	0.238
	Yes	12(10.7%)	21(18.8%)	33(29.5%)		
Tumor factor						
Tumor size	<5 cm	14(12.5%)	40(35.7%)	54(48.2%)	0.358	0.550
	≥5 cm	18(16.1%)	40(35.7%)	58(51.8%)		
Tumor Pathological Grade	High Differentiation	22(19.6%)	7(6.3%)	29(25.9%)	42.883	＜0.001*
	Moderate and Low Differentiation	10(8.9%)	73(65.2%)	83(74.1%)		
TNM Stage	High-risk II	24(21.4%)	14(12.5%)	38(33.9%)	33.712	＜0.001*
	III-IV	8(7.2%)	66(58.9%)	74(66.1%)		
Ki67(%)	＜20%	25(22.3%)	14(12.5%)	39(34.8%)	37.015	＜0.001*
	≥20%	7(6.3%)	66(58.9%)	73(65.2%)		
MMR	dMMR	25(22.3%)	14(12.5%)	39(34.8%)	37.016	＜0.001*
	pMMR	7(6.3%)	66(58.9%)	73(65.2%)		
Venous invasion	No	15(13.4%)	42(37.5%)	57(50.9%)	0.289	0.591
	Yes	17(15.2%)	38(33.9%)	55(49.1%)		
Perineural invasion	No	27(24.1%)	13(11.6%)	40(35.7%)	46.204	＜0.001*
	Yes	5(4.5%)	67(59.8%)	72(64.3%)		
Number of lymph nodes detected	＜10	18(16.1%)	38(33.9%)	56(50.0%)	0.700	0.403
	≥10	14(12.5%)	42(37.5%)	56(50.0%)		
Cancer nodules	0	17(15.2%)	18(16.1%)	35(31.3%)	9.978	＜0.002*
	1~3	15(13.4%)	62(55.3%)	77(68.7%)		
CEA（ng/mL）	≤50	21(18.8%)	18(16.1%)	39(34.9%)	18.730	＜0.001*
	＞50	11(9.8%)	62(55.3%)	73(65.1%)		
CA19-9	≤111	17(15.2%)	43(38.4%)	60(53.6%)	0.004	0.952

（U/ml）						
	＞111	15(13.4%)	37(33.0%)	52(46.4%)		
Neutrophils（*10^9）	1.8~6.3	17(15.2%)	41(36.6%)	58(51.8%)	0.102	0.749
	＜1.8 or ＞6.3	15(13.4%)	39(34.8%)	54(48.2%)		
Hemoglobin（g/L）	120~160	16(14.3%)	43(38.4%)	59(52.7%)	0.129	0.720
	＜120	16(14.3%)	37(33.0%)	53(47.3%)		
lymphocyte（*10^9）	1.5-3.3	17(15.2%)	41(36.6%)	58(51.8%)	0.032	0.858
	＜1.5 or ＞3.3	15(13.4%)	39(34.8%)	54(48.2%)		
Albumin（g/L）	≥35	18(16.1%)	41(36.6%)	59(52.7%)	0.175	0.676
	＜35	14(12.5%)	39(34.8%)	53(47.3%)		
WBC（*10^9）	4.0～10.0	18(16.1%)	42(37.5%)	60(53.6%)	0.129	0.719
	＜4 or ＞10	14(12.5%)	38(33.9%)	52(46.4%)		
PLT（*10^9）	100~300	17(15.2%)	39(34.8%)	56(50.0%)	0.175	0.676
	＜100 or ＞300	15(13.4%)	41(36.6%)	56(50.0%)		
CRP	0~10	16(14.3%)	36(32.1%)	52(46.4%)	0.230	0.632
	＞10	16(14.3%)	44(39.3%)	60(53.6%)		
Recurrence	No	26(23.2%)	31(27.7%)	57(50.9%)	16.520	＜0.001*
	Yes	6(5.4%)	49(43.7%)	55(49.1%)		
Vital Status	Alive	28(25.0%)	44(39.3%)	72(64.3%)	10.516	0.001*
	Dead	4(3.6%)	36(32.1%)	40(35.7%)		

Table S3 Univariate Cox hazard ratio regression analysis of prognosis-CRC patients after oxaliplatin treatment

Characteristics		OS		RFS	
		HR(95%CI)	*P value*	HR(95%CI)	*P value*
Demographics					
Sex	Male	1.0 (ref)	P = 0.591	1.0 (ref)	P = 0.876
	Female	0.84(0.44,1.59)		0.96(0.55,1.67)	
Age (year)	<60	1.0 (ref)	P = 0.181	1.0 (ref)	P = 0.404
	≥60	1.02(0.99,1.06)		1.01(0.98,1.04)	
Diabetes	No	1.0 (ref)	P = 0.608	1.0 (ref)	P = 0.632
	Yes	1.22(0.58,2.55)		1.17(0.62,2.22)	
hypertension	No	1.0 (ref)	P = 0.165	1.0 (ref)	P = 0.444
	Yes	1.58(0.83,2.99)		1.25(0.71,2.19)	
Tumor factor					
Tumor size	<5 cm	1.0 (ref)	P = 0.062	1.0 (ref)	P = 0.108
	≥5 cm	0.55(0.29,1.03)		0.65(0.38,1.10)	
Tumor Pathological Grade	High Differentiation	1.0 (ref)	P = 0.072	1.0 (ref)	P = 0.009*
	Moderate and Low Differentiation	2.22(0.93,5.30)		2.89(1.31,6.40)	
TNM Stage	High-risk II	1.0 (ref)	P = 0.002*	1.0 (ref)	P= 0.002*
	III-IV	4.39(1.72,11.21)		3.01(1.51,5.98)	
Ki67(%)	＜20%	1.0 (ref)	P = 0.024*	1.0 (ref)	P = 0.077
	≥20%	2.44(1.12,5.29)		1.72(0.94,3.11)	
MMR	dMMR	1.0 (ref)	P = 0.003*	1.0 (ref)	P＜0.001*
	pMMR	3.79 (1.59,9.04)		3.94(1.93,8.08)	
Venous invasion	No	1.0 (ref)	P = 0.384	1.0 (ref)	P = 0.985
	Yes	0.76(0.40,1.42)		1.01(0.59,1.71)	
Perineural invasion	No	1.0 (ref)	P = 0.333	1.0 (ref)	P = 0.128
	Yes	1.40(0.71,2.75)		1.57(0.88,2.81)	
ARL4C IHC scores		1.24(1.11,1.39)	P＜0.001*	1.25 (1.14,1.38)	P＜0.001*
Number of lymph nodes detected	＜10	1.0 (ref)	P = 0.171	1.0 (ref)	P = 0.259
	≥10	1.55(0.83,2.90)		1.36(0.80,2.31)	
Cancer nodules	0	1.0 (ref)	P = 0.891	1.0 (ref)	P = 0.402
	1~3	1.05(0.53,2.06)		1.29(0.71,2.34)	
CEA（ng/mL）	≤50	1.0 (ref)	P = 0.379	1.0 (ref)	P = 0.057
	＞50	1.37(0.68,2.74)		1.84(0.98,3.43)	
CA19-9（U/ml）	≤111	1.0 (ref)	P = 0.553	1.0 (ref)	P = 0.524
	＞111	0.83 (0.44,1.55)		0.84(0.49,1.43)	
Neutrophils（*10^9）	1.8~6.3	1.0 (ref)	P = 0.953	1.0 (ref)	P = 0.721
	＜1.8 or ＞6.3	0.98(0.53,1.83)		1.10(0.65,1.87)	
Hemoglobin（g/L）	120~160	1.0 (ref)	P = 0.951	1.0 (ref)	P = 0.994
	＜120	1.02(0.55,1.90)		1.00(0.59,1.69)	
lymphocyte（*10^9）	1.5-3.3	1.0 (ref)	P = 0.065	1.0 (ref)	P = 0.069

	＜1.5 or ＞3.3	1.81(0.96,3.42)		1.64(0.96,2.81)	
Albumin（g/L）	≥35	1.0 (ref)	P = 0.496	1.0 (ref)	P = 0.892
	＜35	1.24(0.67,2.32)		0.96(0.57,1.64)	
WBC（*10^9）	4.0～10.0	1.0 (ref)	P = 0.453	1.0 (ref)	P = 0.791
	＜4 or ＞10	0.79(0.42,1.47)		0.93(0.55,1.58)	
PLT（*10^9）	100~300	1.0 (ref)	P = 0.463	1.0 (ref)	P = 0.954
	＜100 or ＞300	1.26(0.68,2.35)		1.02(0.60,1.72)	
CRP	0~10	1.0 (ref)	P = 0.876	1.0 (ref)	P = 0.790
	＞10	0.95(0.51,1.77)		1.08(0.63,1.83)	

Table S4 Multivariant Cox hazard ratio regression analysis of prognosis-CRC patients after oxaliplatin treatment

Characteristics		OS		RFS	
		HR(95%CI)	*P value*	HR(95%CI)	*P value*
Demographics					
Sex	Male	1.0 (ref)	P = 0.628	1.0 (ref)	P = 0.093
	Female	1.21(0.56,2.64)		1.75(0.91,3.37)	
Age (year)	<60	1.0 (ref)	P = 0.709	1.0 (ref)	P = 0.850
	≥60	1.01(0.96,1.06)		1.01(0.97,1.04)	
Diabetes	No	1.0 (ref)	P = 0.270	1.0 (ref)	P = 0.148
	Yes	1.94(0.60,6.32)		2.10(0.77,5.72)	
hypertension	No	1.0 (ref)	P = 0.094	1.0 (ref)	P = 0.796
	Yes	2.04(0.89,4.71)		1.10(0.53,2.27)	
Tumor factor					
Tumor size	<5 cm	1.0 (ref)	P = 0.782	1.0 (ref)	P = 0.478
	≥5 cm	1.15 (0.43,3.08)		1.33(0.61,2.89)	
Tumor Pathological Grade	High Differentiation	1.0 (ref)	P = 0.288	1.0 (ref)	P = 0.429
	Moderate and Low Differentiation	0.49(0.13,1.82)		0.62(0.19,2.01)	
TNM Stage	High-risk II	1.0 (ref)	P = 0.014*	1.0 (ref)	P = 0.042*
	III-IV	4.08(1.33,12.56)		2.24(1.03,4.89)	
Ki67(%)	＜20%	1.0 (ref)	P = 0.374	1.0 (ref)	P = 0.304
	≥20%	1.67(0.54,5.21)		0.65(0.29,1.48)	
MMR	dMMR	1.0 (ref)	P = 0.040*	1.0 (ref)	P = 0.014*
	pMMR	2.82(1.05,7.55)		2.78(1.23,6.26)	
Venous invasion	No	1.0 (ref)	P = 0.809	1.0 (ref)	P = 0.154
	Yes	0.89(0.35,2.27)		1.73(0.82,3.67)	
Perineural invasion	No	1.0 (ref)	P = 0.290	1.0 (ref)	P = 0.124
	Yes	0.58(0.21,1.60)		0.52(0.23,1.20)	
ARL4C IHC scores		1.24(1.02,1.50)	P = 0.035*	1.35(1.15,1.59)	P＜0.001*
Number of lymph nodes detected	＜10	1.0 (ref)	P = 0.143	1.0 (ref)	P = 0.783
	≥10	2.03(0.79,5.26)		1.11(0.52,2.40)	
Cancer nodules	0	1.0 (ref)	P = 0.540	1.0 (ref)	P = 0.579
	1~3	0.76(0.32,1.82)		0.81(0.38,1.71)	
CEA（ng/mL）	≤50	1.0 (ref)	P = 0.168	1.0 (ref)	P = 0.688
	＞50	0.50(0.19,1.33)		0.85(0.37,1.93)	
CA19-9（U/ml）	≤111	1.0 (ref)	P = 0.369	1.0 (ref)	P = 0.493
	＞111	0.69(0.30,1.56)		0.79(0.41,1.54)	
Neutrophils（*10^9）	1.8~6.3	1.0 (ref)	P = 0.206	1.0 (ref)	P = 0.077
	＜1.8 or ＞6.3	1.83(0.72,4.67)		1.98(0.93,4.24)	
Hemoglobin（g/L）	120~160	1.0 (ref)	P = 0.306	1.0 (ref)	P = 0.966
	＜120	1.53(0.68,3.45)		1.01(0.55,1.86)	
lymphocyte（*10^9）	1.5-3.3	1.0 (ref)	P = 0.174	1.0 (ref)	P = 0.084

	＜1.5 or ＞3.3	1.81(0.77,4.25)		1.90(0.92,3.95)	
Albumin（g/L）	≥35	1.0 (ref)	P = 0.825	1.0 (ref)	P = 0.561
	＜35	1.10 (0.48,2.52)		0.81(0.40,1.64)	
WBC（*10^9）	4.0～10.0	1.0 (ref)	P = 0.127	1.0 (ref)	P = 0.379
	＜4 or ＞10	0.55(0.26,1.18)		0.73(0.36,1.47)	
PLT（*10^9）	100~300	1.0 (ref)	P = 0.148	1.0 (ref)	P = 0.701
	＜100 or ＞300	1.98(0.79,4.97)		0.86(0.40,1.86)	
CRP	0~10	1.0 (ref)	P = 0.142	1.0 (ref)	P = 0.432
	＞10	0.56(0.25,1.22)		0.77(0.41,1.47)	

Table S5: Analysis of molecular docking results between ARL4C and related proteins and β-Lapachone.

1.Illustration of the molecular docking results of ARL4C with related Deubiquitinating Enzymes (DUBs):

In the Figure S6G-K and Figure 5G, the blue ribbon structures indicate ARL4C proteins, and the red ribbon structures indicate USP1, USP35, USP37, USP38, USP42, USP49 proteins. Bar structures indicate interacting amino acid residues. In the three-dimensional diagram, the solid yellow line indicates hydrogen bonding, and in the red and black two-dimensional diagram, the green dashed line indicates hydrogen bonding forces and the blue dashed line indicates salt bridge forces.

1.1 Analysis of ARL4C and USP1 molecular docking results (Figure S6G):

The binding score between ARL4C and USP1 protein docking is -246.12 with a confidence score of 87.24%. Generally speaking, the lower the binding score of two protein docking, the better the binding stability with a confidence score greater than 70%.

The hydrogen bonding and salt bridging between ARL4C(B) and USP1(A) proteins can be seen from the 2D plot as follows:

hydrogen-bond interaction	Salt bridge interaction
Ser568(A) - Gly2(B)	
Glu464(A) - Gln188(B)	Glu469(A) - Arg84(B)
Val495(A) - Lys190(B)	
Glu481(A) - Ser9(B)	
Ser475(A) - His65(B)	

It is due to the above forces that the two proteins can bind stably.

1.2 Analysis of ARL4C and USP35 molecular docking results (Figure S6H):

The binding score between ARL4C and USP35 protein docking is -294.10 with a confidence score of 94.69%. Generally speaking, the lower the binding score of two protein docking, the better the binding stability with confidence score greater than 70%.

The hydrogen bonding and salt bridging between ARL4C(B) and USP35(A) proteins can be seen from the 2D plot as follows：

hydrogen-bond interaction	Salt bridge interaction
Gln63(A) - Lys190(B)	
Glu113(A) - Lys51(B)	
Gln109(A) - Cys85(B)	
Arg150(A) - Glu116(B)	Glu113(A) - His65(B)
Arg70(A) - Ser9(B)	Glu27(A) - Lys191(B)
Arg30(A) - Asn7(B)	
His71(A) - Asn7(B)	
His71(A) - Lys187(B)	
Leu110(A) - Gln12(B)	

It is due to the above forces that the two proteins can bind stably.

1.3 Analysis of ARL4C and USP37 molecular docking results (Figure S6I):

The binding score between ARL4C and USP37 protein docking is -256.54 with a confidence score

of 89.38%. Generally speaking, the lower the binding score of two protein docking, the better the binding stability with confidence score greater than 70%.

The hydrogen bonding and salt bridging between ARL4C(B) and USP37(A) proteins can be seen from the 2D plot as follows：

hydrogen-bond interaction	Salt bridge interaction
Ser415(A) - Gln12(B)	Glu419(A) - Lys187(B)
Ser415(A) - Lys187(B)	
Lys446(A) - Cys85(B)	
Thr450(A) - Ser81(B)	
Gln247(A) - Gly2(B)	

It is due to the above forces that the two proteins can bind stably.

1.4 Analysis of ARL4C and USP38 molecular docking results (Figure 5G):

The binding score between ARL4C and USP38 protein docking was -275.69 with a confidence score of 92.51%. Generally speaking, the lower the binding score of two protein docking, the better the binding stability with a confidence score greater than 70%.

The hydrogen bonding between ARL4C(B) and USP38(A) proteins can be seen from the 2D plot as follows:

hydrogen-bond interaction
Cys180(A) - Asn40(B)
Glu185(A) - Ser23(B)
Thr183(A) - Lys128(B)
Thr183(A) - Ser23(B)
Ser934(A) - Phe11(B)

It is due to the above forces that the two proteins can bind stably.

1.5 Analysis of ARL4C and USP42 molecular docking results (Figure S6J):

The binding score between ARL4C and USP42 protein docking is -239.34 with a confidence score of 85.65%. Generally speaking, the lower the binding score of two protein docking, the better the binding stability with a confidence score greater than 70%.

The hydrogen bonding and salt bridging between ARL4C(B) and USP42(A) proteins can be seen from the 2D plot as follows:

hydrogen-bond interaction	Salt bridge interaction
Asp388(A) - Arg84(B)	Asp331(A) - Lys51(B)
Gln385(A) - Tyr82(B)	
Ser392(A) - Tyr82(B)	
Ser398(A) - Thr49(B)	
Ser398(A) - Gln402(A)	
Ser401(A) - Lys51(B)	
Lys327(A) - Gln12(B)	

It is due to the above forces that the two proteins can bind stably.

1.6 Analysis of ARL4C and USP49 molecular docking results (Figure S6K):

The binding score between ARL4C and USP49 protein docking was -264.18 with a confidence score of 90.75%. In general, the lower the binding score of two protein dockings with a confidence score greater than 70%, the better the binding stability.

The hydrogen bonding and salt bridging between ARL4C(B) and USP49(A) proteins can be seen

from the 2D plot as follows:

hydrogen-bond interaction	Salt bridge interaction
Glu360(A) - Lys191(B)	
Ser377(A) - Lys190(B)	
Asp86(A) - Asp87(B)	
Asp86(A) - Arg84(B)	Asp86(A) - Arg84(B)
Arg147(A) - Gln12(B)	
Pro88(A) - Ser81(B)	
Gln36(A) - Ala115(B)	
Glu30(A) - Lys113(B)	

It is due to the above forces that the two proteins can bind stably.

2.Analysis of molecular docking results of ARL4C protein and RAC1 protein (Figure 6C, Figure S8A):

In the three-dimensional plots, bar structures indicate interacting amino acid residues, yellow dashed lines indicate hydrogen bonds, blue ribbon structures indicate ARL4C proteins, and green ribbon structures indicate RAC1 proteins. In the red and black two-dimensional diagrams, the red dashed lines indicate salt-bridge forces and the green dashed lines indicate hydrogen-bonding forces. In general, the lower the binding scores of two proteins docked, the better the binding stability, which corresponds to the higher confidence level, the stronger the binding stability. In the docking result the docking score is -227.23 confidence level is 0.8241, when the confidence level is greater than 0.7 is responsible for stable binding.

As can be seen from the three-dimensional diagram, nine hydrogen bonds were formed by docking, which were amino acid residue 43 of ASN of ARL4C protein and amino acid residue 190 of LYS of RAC1 protein, amino acid residue 25 of THR of ARL4C protein and amino acid residue 82 of TYR of RAC1 protein, amino acid residue 26 of ASN of ARL4C protein and amino acid residue 84 of RAC1 protein, amino acid residue 1 of ARG of ARL4C protein and amino acid residue 1 of RAC1 protein, and amino acid residue 1 of RAC1 protein and amino acid residue 3 of RAC1 protein and amino acid residue 4 of RAC1 protein. ARG amino acid residue, SER amino acid residue #41 of the ARL4C protein with GLN amino acid residue #12 of the RAC1 protein, ASN amino acid residue #39 of the ARL4C protein with PHE amino acid residue #11 of the RAC1 protein, and TYR amino acid residue #40 of the ARL4C protein with SER amino acid residue #13 of the RAC1 protein, ASP amino acid residue #38 of the ARL4C protein with amino acid residue #51 LYS of the RAC1 protein, and VAL amino acid residue #36 of the ARL4C protein with amino acid residue #51 LYS of the RAC1 protein.

It is due to the presence of the above forces that the ARL4C protein and the RAC1 protein can be stably bound.

3.Analysis of molecular docking results of ARL4C protein and RAP1A protein (Figure 6D, Figure S8B):

In the three-dimensional diagrams, bar structures indicate interacting amino acid residues, yellow dashed lines indicate hydrogen bonds, blue ribbon structures indicate ARL4C proteins, and green ribbon structures indicate RAP1A proteins. In the red and black two-dimensional diagrams, the red dashed line indicates salt-bridge water forces and the green dashed line indicates hydrogen

bonding forces. In general, the lower the binding score of two proteins docked, the better the binding stability, corresponding to the higher confidence level, the stronger the binding stability. In the docking result the docking score is -208.64 confidence level is 0.7637, when the confidence level is greater than 0.7 is responsible for stable binding.

As can be seen from the three-dimensional diagram, 13 hydrogen bonds were formed by docking, which were amino acid residue 133 ASN of ARL4C protein with amino acid residues 99 ASP and 97 ASP of RAP1A protein, amino acid residue 137 GLN of ARL4C protein with amino acid residues 23 SER and 100 ARG of RAP1A protein, amino acid residue 94 GLN of ARL4C protein with amino acid residues 23 ASP and 97 ASP of RAP1A protein, and amino acid residue 94 GLN of ARL4C protein with amino acid residues 23 ASP and 100 ARG of RAP1A protein, and amino acid residue 94 ASP of ARL4C protein. GLN amino acid residue with amino acid residue #100 ARG of the RAP1A protein, amino acid residue #95 ASP of the ARL4C protein with amino acid residue #48 ASN of the RAP1A protein, amino acid residue #97 ARG of the ARL4C protein with amino acid residue #23 SER of the RAP1A protein, and amino acid residue #98 GLU of the ARL4C protein with the SER #23 of the RAP1A protein, amino acid residue #27 of THR, amino acid residue #107 of GLU of the ARL4C protein with amino acid residue #28 of THR of the RAP1A protein, and amino acid residue #105 of ASP of the ARL4C protein with amino acid residue #32 of ARG of the RAP1A protein.

It is due to the presence of the above forces that the ARL4C protein and the RAP1A protein can be stably bound.

4. Analysis of the results of molecular docking of ARL4C with β-Lapachone (Figure 8A):

As can be seen from the three-dimensional diagram, the yellow structure is the compound structure and the green structure is the ARL4C amino acid residue. 2 hydrogen bonds were formed by docking, which are amino acid residue 127 ASN and amino acid residue 28 THR of ARL4C protein connected to the compound. Docking formed 3 hydrophobic bonds, amino acid residue 128 LYS, amino acid residue 163 ILE of ARL4C protein connected to the compound. Blue thin line hydrogen bond, gray hydrophobic bond. Blue thick nitrogen atom, red thick oxygen atom, ASN-2 is the binding site, 127,28 hydrogen bonding site, 128 163 is the hydrophobic bonding site. This force results in a relatively low binding energy and stable binding, with a binding energy of -7.3 (kcal/mol), which is less than -5 to stabilize the binding.
